# Supplementary material for: Realist inquiry into Maternity care @ a Distance (ARM@DA): realist review protocol
Source: BMJ Open. 2022 Sep 20;12(9):e062106. doi: 10.1136/bmjopen-2022-062106 (PMC9490633; doi:10.1136/bmjopen-2022-062106)

Supplemental File 1: Digital Clinical Consultation in Maternity Care: Realist Review Protocol Process Flowchart

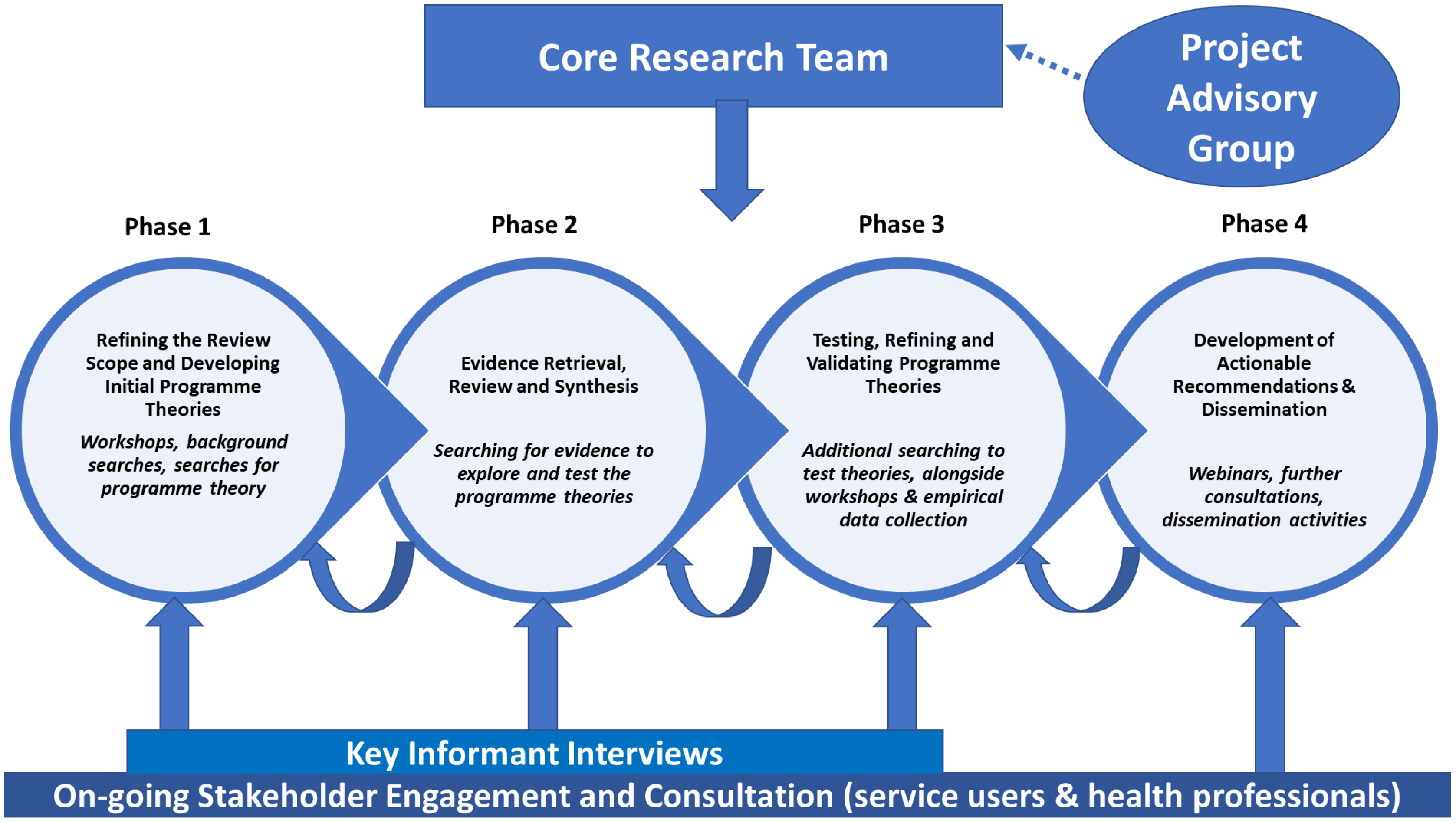

Supplement: Supplementary data [file bmjopen-2022-062106supp001.pdf]
