# Supplementary material for: Realist inquiry into Maternity care @ a Distance (ARM@DA): realist review protocol
Source: BMJ Open. 2022 Sep 20;12(9):e062106. doi: 10.1136/bmjopen-2022-062106 (PMC9490633; doi:10.1136/bmjopen-2022-062106)
Supplement: Author's manuscript [file bmjopen-2022-062106.draft_revisions.pdf]

BMJ Open is committed to open peer review. As part of this commitment we make the peer review history of every article we publish publicly available.

When an article is published we post the peer reviewers' comments and the authors' responses online. We also post the versions of the paper that were used during peer review. These are the versions that the peer review comments apply to.

The versions of the paper that follow are the versions that were submitted during the peer review process. They are not the versions of record or the final published versions. They should not be cited or distributed as the published version of this manuscript.

BMJ Open is an open access journal and the full, final, typeset and author-corrected version of record of the manuscript is available on our site with no access controls, subscription charges or pay-per-view fees (http://bmjopen.bmj.com).

If you have any questions on BMJ Open's open peer review process please email info.bmjopen@bmj.com

BMJ Open

A Realist Inquiry into Maternity Care @ a Distance (ARM@DA): Realist Review Protocol

Journal:	*BMJ Open*
Manuscript ID	bmjopen-2022-062106
Article Type:	Protocol
Date Submitted by the Author:	17-Feb-2022
Complete List of Authors:	Evans, Catrin; University of Nottingham, School of Health Sciences,<br>Evans , Kerry; University of Nottingham, School of Health Sciences,<br>Booth, Andrew; The University of Sheffield, ScHARR<br>Timmons, Stephen; University of Nottingham, Business School<br>Jones, Nia; University of Nottingham, School of Medicine<br>Sunney, Candice ; Nottingham Maternity Research Network<br>Nazmeen, Benash; University of Nottingham, School of Health Sciences,<br>Clowes, Mark; The University of Sheffield, ScHARR<br>Spiby, Helen; University of Nottingham, School of Health Sciences
Keywords:	Organisation of health services < HEALTH SERVICES ADMINISTRATION & MANAGEMENT, Maternal medicine < OBSTETRICS, QUALITATIVE RESEARCH, STATISTICS & RESEARCH METHODS, Telemedicine < BIOTECHNOLOGY & BIOINFORMATICS

SCHOLARONE™
Manuscripts

A Realist Inquiry into Maternity Care @ a Distance (ARM@DA): Realist Review Protocol

- Catrin Evans [*Corresponding Author*], School of Health Sciences, University of Nottingham, Nottingham, U.K. NG7 2UH, catrin.evans@nottingham.ac.uk, Tel. +44 115 823 0894; ORCID: 0000-0002-5338-2191
- Kerry Evans, School of Health Sciences, University of Nottingham, Nottingham, UK
- Andrew Booth, School of Health and Related Research, University of Sheffield, Sheffield, UK
- Stephen Timmons, Business School, University of Nottingham, Nottingham, UK
- Nia Jones, School of Medicine, University of Nottingham, Nottingham, UK
- Candice Sunney, Nottingham Maternity Research Network, Nottingham, UK
- Benash Nazmeen, School of Health Sciences, University of Nottingham, Nottingham, UK
- Mark Clowes, School of Health and Related Research, University of Sheffield, Sheffield, UK
- Helen Spiby, School of Health Sciences, University of Nottingham, Nottingham, UK

Keywords

- realist review; remote care; digital clinical consultation; virtual consultation; tele-health;

Word Count

4,612

For peer review only

ABSTRACT

INTRODUCTION

One of the most commonly reported Covid-19-related changes to all maternity services has been an increase in the use of digital clinical consultations such as telephone or video-calling. Remote/virtual consultations are increasingly widespread, however the ways in which they can be optimally utilised along maternity care pathways remains unclear. In particular, it is imperative that digital service innovations do not further exacerbate (and, ideally, should tackle) existing inequalities in service access and clinical outcomes. This project aims to undertake an in-depth and theory-informed investigation of the evidence around implementation of digital clinical consultations. It seeks to illuminate how digital consultations can work to support safe, personalised and appropriate maternity care and to clarify when they might be most appropriately used, for whom, when, and in what contexts?

METHODS AND ANALYSIS

A realist synthesis will be conducted in 4 iterative phases, with stakeholder involvement (service users and health professionals) embedded throughout: (i) refining the review focus and generating initial programme theories, (ii) exploring and developing the programme theories in light of evidence, (iii) testing/refining the programme theories, and, (iv) constructing actionable recommendations in ways that can be rapidly adopted into practice and clearly define a future research agenda. The review will draw upon three sources of evidence: (i) literature (published and unpublished research, audit, evaluation and theory), (ii) stakeholder expertise and insights, and (iii) key informant interviews. The review will follow the RAMESES (Realist and Meta-narrative Evidence Synthesis Evolving Standards) quality procedures and reporting guidance.

ETHICS AND DISSEMINATION

Ethical approval has been obtained from the University of Nottingham, Faculty of Medicine and Health Sciences Ethics Committee (FMHS 426-1221). The review findings will be disseminated in a range of formats relevant to different audiences, including reports/publications, webinars, and an online education resource.

PROSPERO REGISTRATION

No. CRD42021288702

For peer review only - http://bmjopen.bmj.com/site/about/guidelines.xhtml

STRENGTHS AND LIMITATIONS OF THIS STUDY

- A realist synthesis moves beyond effectiveness and acceptability to consider evidence related to implementation (what works, for whom and under what contexts)
- The evidence based around digital/remote consultations is fast-moving. The iterative approach to searching will enable relevant evidence to be found, even after the initial searches have taken place
- Stakeholder engagement (service users and health professionals) is embedded at every stage of the review
- The review team is UK-based and the project recommendations will be oriented to the UK National Health Service (NHS). However, the review will include international evidence and its focus on mechanisms underlying implementation will yield insights that are potentially transferable across geographical and clinical settings

For peer review only

INTRODUCTION

This article sets out a protocol for a realist review to generate a theory- and evidence-informed framework to guide best practice in the implementation of digital clinical consultations in maternity care. The project is being undertaken by a UK-based team and is oriented to producing findings that are actionable in the context of the UK's National Health Service (NHS). Nonetheless, the project will draw upon evidence from comparable geographical and health system settings and hence its findings will be of international relevance.

Maternity care in England is undergoing a substantive transformation programme whose overall aim is to support services to work across professional and service boundaries to become safer, kinder, more personalised, and more family-friendly.[1-3] A core work stream is 'harnessing digital technology'.[4, 5] In the UK, as in many other countries, the Covid-19 pandemic has radically altered and dramatically accelerated the context of this digital policy imperative.[6-9]

One of the most commonly reported Covid-related changes to all forms of service provision in maternity care has been an increase in the use of remote/virtual consultations using telephone or video.[4, 7, 10-18] For example, one recent survey of all 194 obstetric units in England (42% response rate) found that 89% reported using digitally delivered consultation methods in antenatal care and 56.8% for postnatal care.[7] For antenatal services, the majority of these (87.7%) were conducted via the telephone whereas a smaller percentage were conducted using everyday video technology or specialist video technology (12% and 25.9% respectively). A UK survey of 524 women in 2020 reported that 51.8% had experienced telephone or video consultations.[19]

Within maternity care, consultations have a wide range of potential purposes depending upon the individual circumstances and characteristics of the service user, clinical categorisation of the pregnancy (e.g. high risk or low risk) and stage of the maternity care pathway (e.g. antenatal care, assessment of early labour, postnatal care). Consultations may involve clinical (physical and mental health) assessment, monitoring, safeguarding assessments, health promotion, information giving, education or therapeutic support. The interactions need to be organised and implemented within multi-professional care pathways that seek to promote continuity, personalisation and choice, recognise diversity and ensure safety.[9] Likewise, consultations need to be supported by auditable records that can be accessed by, and strengthen communications between, different providers and settings.

The rapid shift to remote or digitally delivered clinical consultations was initially implemented to support pandemic response objectives such as social distancing and service demand management.[6, 9, 16, 20-22] Going forward however, a key challenge for maternity services lies in how to 're-purpose' the use of remote consultations to serve longer term quality, equity and productivity objectives.[6, 9] Although remote consultations may now be widespread, the ways in which they can optimally be utilised in future along maternity care pathways remains unclear. In particular, it is imperative that service innovations do not further exacerbate (and, ideally, should tackle) existing inequalities in service access and clinical outcomes.[9, 14, 23, 24]

For peer review only - http://bmjopen.bmj.com/site/about/guidelines.xhtml

Hence, there is a need for a comprehensive review of the evidence base to inform future service developments and future research.[6]

Operational Definitions

Digitally-facilitated clinical communication has a highly diverse nomenclature, including terms such as telehealth, telemedicine, virtual/remote care, video consultations and digital consultation (amongst many others). In spite of efforts by WHO in 2017 to standardise concepts and terminology, there is no internationally agreed or consistent set of terms, meanings or definitions.[25] A similar diversity is found in the rapidly evolving technologies and systems used to implement digitally-facilitated consultations. Key distinctions are that digital technologies can be utilised for: (i) direct 'live' clinical communication/consultation between a service user and a practitioner (synchronous), or (ii) direct communication that may happen at different time points (asynchronous), such as text messaging. This is in contrast to other digital care modalities such as remote monitoring systems (e.g. remote blood pressure monitoring), on-line triage algorithms, the use of apps, wearable personal devices or electronic medical records which are usually asynchronous and do not involve direct interpersonal patient/practitioner communication (although these may be utilised to trigger, or to directly inform, consultations).

The focus of this project is on maternity care **consultations** that are facilitated through, and/or complemented by, digital technology. To capture this focus, we will use the term 'Digital Clinical Consultation' – henceforth referred to as 'DC-CON'.[26] The working definition for our project is outlined in Table 1:

Table 1: Operational Definition

Digital Clinical Consultation (DC-CON) *– Synchronous telephone or video consultations involving direct interaction between a service user and a maternity healthcare professional. It has two-way functionality and can be initiated by either party. It may be linked to, or complemented by, other digital technologies within the maternity care pathways.*

This definition recognises that the focus is on the consultation itself, but that this might be linked to, or informed by, other digital technologies.

This project will investigate digital consultations across the maternity pathway, i.e. during pregnancy, the intrapartum period and early postnatal care (up to 10-14 days post-partum).

Existing Evidence on DC-CON in Maternity Care

There is a large and rapidly expanding literature associated with DC-CON in maternity care.[20] For simplicity, we conceptualise the purpose of DC-CON in two ways (while recognising significant overlap between the two). The first relates to DC-CON that is additional to usual care – where specialist support is required on a single issue where a specific need is identified (e.g. interventions to support perinatal mental health, breastfeeding or smoking cessation). There is now substantial evidence that such targeted interventions can be feasible, acceptable and effective to varying degrees.[20, 27-33]

For peer review only - http://bmjopen.bmj.com/site/about/guidelines.xhtml

The second DC-CON purpose relates to situations where DC-CON is already the main modality of care (e.g. in telephone helplines or triage systems).[34] It also refers to situations where standard care pathways are altered so that some in-person points of contact are replaced or supplemented by DC-CON (as happened during the Covid-19 pandemic). DC-CON has been investigated with regard to antenatal care among both high[35, 36] and low[37, 38] risk women. For example, a recent randomised control trial in the USA sought to compare standard in-person antenatal care with a new 'hybrid' system that included virtual consultations. This study found higher satisfaction with care, no difference in health outcomes and less anxiety in the hybrid care group.[37] However, a systematic review of telephone support for women in pregnancy and postpartum was less conclusive, with results suggesting that telephone support may be associated with higher overall satisfaction with care while yielding uncertain impacts on clinical outcomes.[30]

Overall, existing research on DC-CON in maternity care shows it can be safe and acceptable in controlled conditions.[27-29, 35, 38-40].[37, 41] Research also shows that the experiences of staff and women with DC-CON vary quite significantly[42].[7, 12, 34, 39, 43-50] A gap remains therefore in understanding the conditions required for safe and acceptable DC-CON implementation and in understanding the factors that underpin variation in experience. Such variation is problematic, as it is implementation in real-world contexts, at scale, that needs to be understood for the systems-wide transformation as envisaged in the NHS Maternity Transformation Programme.[2, 21, 51] It is this gap that the proposed project will address.

Research Aim and Question

This project aims to undertake an in-depth and theory informed investigation of the evidence around implementation of digital clinical consultations. It seeks to illuminate how digital consultations can work to support safe, personalised and appropriate maternity care and to clarify when they might be most appropriately used, for whom, when, and in what contexts.

Scoping searches of PROSPERO, Medline and the Cochrane Library have not identified any similar reviews currently being undertaken.

PHILOSOPHY AND METHODOLOGY

We will undertake a realist synthesis in which PPI and diverse stakeholder participation is embedded at every stage.[52] Realist syntheses seek to investigate the relationships between what can be observed and experienced, human interpretation of this reality and 'unseen' underlying social structures.[53-55] Increasingly, realist approaches are applied in health research to investigate the complexity of health-related behaviour and to explore how behaviours are shaped by human agency via intersecting social structures that manifest differently in different contexts.[56] Realist approaches are particularly well suited to investigating complex interventions such as DC-CON.[57, 58] This is because realist inquiry seeks to establish causal relationships - expressed as 'programme theories' - between intersecting intervention components, contexts and outcomes.[59] When applied to evidence synthesis, a realist approach focuses on understanding *how* particular interventions lead to particular outcomes under particular contexts (i.e. 'how does A lead to B'? and 'how might B be affected when A is implemented through contexts C, D or E'?).[60] This contrasts with the linear or deterministic approaches adopted by conventional systematic reviews which ask:

For peer review only - http://bmjopen.bmj.com/site/about/guidelines.xhtml

'does A lead to B'? (e.g. an effectiveness review) or: 'is A acceptable or meaningful to a particular group of individuals in a particular context'? (e.g. a qualitative review).[52]

As applied to our research question, the logic of a realist review proposes that different types of interventions (e.g. a video call or a phone call) supply particular resources into a situation that prompt diverse possible reactions and responses (also referred to as 'reasonings') from women and health professionals.[61] The interaction between the resource and the response constitutes a 'mechanism.'[61] Mechanisms are influenced by differences in how an intervention is delivered but also by the context of particular women's lives or different service configurations.[61] This means that the acceptability and outcomes of DC-CON may be highly contextually contingent.[62] A realist approach seeks to identify certain patterns ('demi-regularities'[53]) between types of resource (e.g. a phone call), how individuals respond and how a particular context may alter these responses. Hence, a realist review is focused on identifying and testing programme theories that can account for the contingent nature of intervention implementation.[63] These mid-range explanatory theories are a principal output from a realist review. Their insights make a substantive contribution to health service innovation because, by taking context into account, they are potentially 'transferable' – producing insights relevant to a wide range of settings and geographical contexts.[64]

Our proposed realist synthesis will be conducted in 4 iterative phases (see below). The review will follow the RAMESES (Realist and Meta-narrative Evidence Synthesis Evolving Standards) quality procedures[63] and, subsequently, will comply with RAMESES reporting guidance.[65]

Each phase of the review potentially draws upon three sources of evidence: (i) literature (published and unpublished research, audit, evaluation and theory), (ii) diverse stakeholder expertise and insights, and (iii) key informant interviews.

PATIENT AND PUBLIC INVOLVEMENT

This project has been informed by service user involvement from its outset. A maternity research priority setting exercise was undertaken in 2020 by some members of the research team in which a PPI research network played a key role (the Nottingham Maternity Research Network - NMRN).[66] DC-CON was identified as an important topic for future work. A member of the NMRN (CS) is a co-investigator on the current project, and several other NMRN members are part of a 'Community and Service User Stakeholder Group' (COSU-SG) which will inform all project phases. Other COSU-SG participants include service users and representatives from organisations advocating for quality care and women's rights in pregnancy (Women and Family Health Services and the National Autistic Society).

In addition to the COSU-SG, all project phases will also be informed by a health professional stakeholder group (HP-SG) which includes midwives and obstetricians.

METHODS AND ANALYSIS

Overarching Approach to Literature Searching

For peer review only - http://bmjopen.bmj.com/site/about/guidelines.xhtml

Within this realist review, the overall search approach will follow the only published systematic approach to the “realist search”.[67, 68] This approach extends and enhances the Task and Time Template for a realist review advanced by Pawson.[53] It outlines 4 separate and distinct phases of searching using different retrieval techniques and targeted at different evidence bases, “topped” and “tailed” by precise question formulation and meticulous documentation.[67]

The search strategies within each phase will be developed and operationalised by experienced information specialists (AB & MC). The search dates will be restricted to papers from 2010 to the present to reflect the need for contemporary data. Likewise, inclusion of evidence will be limited to papers in the English language and in high-income settings (OECD countries). The search approach will engage the project advisory group and stakeholders throughout, consulting them where required to provide advice (for example clarifying the terminology used at service level) and help with identifying relevant literature (including, for example, cascading requests to identify literature and policy/practice documents through their associated social media sites and websites).

Key Informant Interviews

Data sources for the project may also involve a small number of key informant interviews. Within a realist review, the purpose of these is to provide additional insights to confirm, refute or refine proposed programme theories.[52] These are most likely to be part of Phase 3 (see below) but may also take place in the other phases if additional data is required to support programme theory development. Potential interviewees may include health professionals or service users. They will be recruited through social media and professional networks with the aim of providing new insights or testing the transferability of programme theories. Interviews will be undertaken remotely, audio-recorded and analysed using a framework approach,[69] mapping the data to the CIMO configurations.

Review Phases

Each phase of the review (with its associated search approaches) is described sequentially below, although in practice there is considerable iteration between them (see Supplemental File 1 for a flow chart of the proposed process).

Phase 1: Refining the Review Scope and Developing Initial Programme Theories

This phase aims to identify and make explicit (through CIMO configurations) an initial set of programme theories that may explain how DC-CON in maternity care can be utilised to achieve optimal outcomes, for whom and in what contexts? Given the potential variability of modality and use of DC-CON in maternity care, an important part of this process is to focus and prioritise the most important questions and outcomes.

Phase 1 involves undertaking some broad background searches to assess the breadth, depth and range of evidence available. The team will then seek to identify key papers (of any study design) on DC-CON implementation that can yield insights for programme theory

For peer review only - http://bmjopen.bmj.com/site/about/guidelines.xhtml

development. The search is iterative, utilising searches on electronic databases, grey literature sources, suggestions from stakeholders, citation tracking and reference list searching of conceptually rich index papers. This stage will include, but will not be restricted to, maternity care settings as we know from pre-protocol scoping that relevant theories have been developed regarding the implementation of DC-CON in other clinical settings.[26, 70-72] Each included paper will be scrutinised to elucidate how 'best practice' in DC-CON is defined and to identify the mechanisms through which successful consultations are purported to work in relation to different contextual configurations and population groups. Details of key theories ('candidate theories') that have been used to explain implementation mechanisms will be extracted. Excel will be used to extract the key characteristics of each paper and NVivo will be used to code the key findings related to implementation. Where possible, findings will be coded and analysed in relation to possible context, mechanism and outcome configurations, helping to generate initial ideas around relevant programme theories. The coding and analysis templates for the reviews in this stage will be developed and piloted by several members of the review team. The majority of the coding will be undertaken by one reviewer, with a second reviewer completing a random sample of approximately 20% to monitor quality and consistency. The findings of this stage will be presented using tables, figures, flow charts and narrative summaries to highlight the key features of the evidence and to describe potential programme theories.

Phase 1 includes two stakeholder workshops (at the beginning and end) to help generate initial programme theories and to refine the key focus of the review.

Phase 2: Evidence Retrieval, Review and Synthesis

The aim of Phase 2 is to determine whether the initial programme theories are supported by empirical evidence and to analyse this evidence to elaborate, refine, adjust and test the theories. This Phase continues the iterative process of literature searching, data extraction and analysis. A core focus is to search for empirical studies that can provide data with which to explore and elaborate the initial programme theories. The search strategy will be iterative, proceeding with carefully formulated searches based on sub-sets of literature constructed using terms associated with the initial programme theories (CIMO frameworks) and key concepts.[67, 73] Searches are initially broad but may then be narrowed down to focus more specifically on evidence associated with particular mechanisms. As an example, in our review, we may construct an initial search using sets of terms derived from our programme theories combined using the 'AND' Boolean operator:

- Context (from the initial programme theory) – postnatal care
- Interventions/phenomenon of interest (from the initial programme theory) – video-based consultation

A follow up search might then focus more specifically on the influence of a mechanism (e.g. trust) in relation to an outcome (e.g. positive health behaviours). In the first instance, the main focus of the search for evidence will be for literature related directly to maternity settings. However, in a realist review, the focus of analysis is the programme theory (or mechanism of action) – hence we may also draw upon wider literature to seek opportunities for transferable learning. In the example above, we might, therefore, seek evidence related

For peer review only - http://bmjopen.bmj.com/site/about/guidelines.xhtml

to 'trust' in the context of video consultations from other clinical settings such as primary care to confirm or refute our emerging theories.

The searches will include systematic reviews and empirical research of any study design, including service evaluation, audit and quality improvement projects. The searches will also include existing policies and practice guidelines surrounding DC-CON in maternity care in the UK. This is because the recommendations set out in policy or practice guidance rest upon implicit or explicit theoretical assumptions regarding implementation. Moreover, the inclusion of policy documents will help to ensure that governance issues are considered alongside implementation so that the review is appropriately contextualised.

As in Phase 1, search sources will include electronic databases, grey literature and expert stakeholders (see Table 2 for sources of evidence and Supplementary File 2 for an initial search strategy for OVID MEDLINE – which will be adapted for different databases). Additional search approaches will include reference list searching, citation tracking, identification of sibling papers (linked papers from a single study) as well as cluster searching[73] which involves building up rich 'cases' of different models of DC-CON in order to grow a cluster of related reports around named or identifiable initiatives to offer both richness and detail. Unlike a conventional systematic review search, searches in a realist review are not necessarily exhaustive but follow the principles of theoretical saturation, ceasing when programme theories are deemed to be sufficiently explained, supported or refuted by the empirical evidence.[63, 67] Likewise, additional targeted searches may be undertaken to explore new mechanisms or other aspects of programme theory that may be identified during the review.[63, 67]

Table 2: Sources of Evidence

Electronic Databases (2010-present)	Grey Literature Sources	Stakeholders
• Cochrane Central Register of Controlled Trials<br>• Cochrane Database of Systematic Reviews<br>• JBI Library<br>• MEDLINE Ovid<br>• Embase Ovid<br>• PsycINFO Ovid<br>• ASSIA Cambridge Scientific Abstracts (Applied Social Sciences Index and Abstracts)<br>• CINAHL EBCSCOhost (Cumulative Index to Nursing and Allied Health Literature)<br>• MIDIRS Ovid	• Google Scholar<br>• Websites (e.g. RCOG, RCM, RCN, NCT, NHS Trusts, NHSX, Health Foundation, WHO)<br>• Conference proceedings<br>• OpenGrey<br>• ProQuest Dissertations & Theses<br>• EThOS – British Library Electronic Theses Online	• Project Advisory Group<br>• Stakeholder groups<br>• Others (e.g. via social media requests, email list serves)

Records will be imported into EndNote and duplicates removed. Study screening and selection will be undertaken by two reviewers independently, with recourse to other team members in cases of ambiguity or disagreement. To aid transparency and consistency, once the initial programme theories have been formulated, an 'inclusion criteria flow chart' will be constructed in which key concepts are operationalised and against which each potential study can be assessed.[74]

For peer review only - http://bmjopen.bmj.com/site/about/guidelines.xhtml

Records will initially be screened by title and abstract. All seemingly relevant papers will then be examined in full-text and reasons for exclusion noted in a table. In line with realist methodology, records will be screened for inclusion based on relevance, rigour and richness.[52, 63] An assessment of relevance considers the extent to which a paper can directly contribute to theory building or theory testing.[63] An assessment of rigour considers whether the methods used to generate the relevant data are credible and trustworthy. Richness refers to the extent to which study findings are fully elaborated through ‘thick description’, grounded in the data and provide explanatory insights.[75] In a realist review, the process of quality assessment refers to the specific data that is relevant to the particular programme theory being examined rather than on the basis of a global evaluation of overall study quality.[76] Hence for each key finding, a judgment needs to be made about the plausibility and coherence of the methods used to generate it.[65] For each included paper, the team will follow the process outlined by Rycroft-Malone *et al*,[60] asking: “*is the evidence provided in this theory area good enough and relevant enough to be included?*”. These judgements will be articulated and recorded for each study as part of the screening and data extraction process.

Data will be extracted from the included studies in two ways. First, information about study characteristics will be extracted into a summary table (as is the case with a conventional systematic review). This will include information on features such as study setting, design, methods, details of intervention modality and technology, participants, outcomes and stage of maternity care pathway. Second, a bespoke data extraction form will be developed based on the initial programme theories, and, as noted above, will include sections in which to note assessments of relevance, richness and rigour. Theory-based data extraction enables the coding and charting of relevant data so that elements of the theory can become populated to investigate what works, for whom, how and in what contexts. The analytical process involves both deductive and inductive coding. Deductive coding will involve extracting data that appears to be directly related to aspects of the programme theory. Where it is possible to make relevant inferences, the data will also be coded in relation to contexts, mechanisms or outcomes. However, the evidence may also reveal new contexts, mechanisms or outcomes which will be identified and coded inductively.

The data extraction templates and associated analytical process will be developed collaboratively amongst the core research team and piloted extensively. Once the team feel they have achieved a clear and consistent approach, the remaining data extraction will be undertaken by one reviewer, with a second reviewer checking approximately 20%. The outputs of this stage will be a set of evidence tables. There will be one overarching table representing the key characteristics of all the studies included in the review. There will then be a series of tables organised to represent the different bodies of literature and findings related to each initial programme theory. Thus, each theory area will have its own evidence table.

The process of data analysis will be ongoing and iterative. The evidence will be reviewed within and across the theory areas to explore how it builds upon, refutes or provides alternative explanations for the initial CIMO configurations. The analytical process will involve asking questions such as: “*What does this evidence suggest about this aspect of our theory? Does it support it? Does it disprove it? Does it suggest an amendment to it*?”[63] This analytic process involves both abductive and retroductive reasoning – i.e. making new

For peer review only - http://bmjopen.bmj.com/site/about/guidelines.xhtml

observations from the evidence, inferring plausible explanations related to the programme theory, seeking to understand the cause of perceived events beyond what can be observed and seeking to identify over-arching patterns. Wong *et al*[63] propose a series of 'conceptual tools' (derived from Pawson, 2005[53]) which will be employed by the review team in this complex process, as indicated in Table 3 below.

Table 3: Analytic Process of a Realist Review

- Juxtaposition of sources of evidence—for example, where evidence about outcomes in one study allows insights into evidence about outcomes in another study;
- Reconciling of sources of evidence—where results differ in comparable circumstances, these will be examined further to find possible reasons for the different results;
- Adjudication of sources of evidence—based on methodological strengths or weaknesses;
- Consolidation of sources of evidence—where outcomes differ in particular contexts, an explanation will be constructed on how and why these outcomes occur differently;
- Situating sources of evidence—when outcomes are different in particular contexts, a possible explanation will be developed as to why they differ

Our stakeholder groups will continue to be consulted at different points to seek their views and test out new ideas.

An important feature of the analysis will be to explore the issue of temporality and sustainability within the evidence. For example, it might be expected that evidence on Covid-19-related DC-CON implementation might include different experiences depending upon the stage of the pandemic and the length of time that staff and service users have had to adjust to changes in service delivery models. Likewise, even in non-Covid-related studies, experiences with new technologies vary depending upon the length of time since introduction, with many studies focusing upon the early stages of implementation rather than exploring how and why technologies become embedded, normalised or sustainable.[72, 77]

In this project, we will endeavour to take temporality into account in two ways. The first, relates to evidence that is specifically concerned with changes made during the Covid-19 pandemic. Here, we will ensure that our data extraction template explicitly acknowledges the time period of data collection, extracting and mapping data to the stages of the pandemic and then transparently considering these stages as part of the analysis. Likewise, working with our advisory and stakeholder groups, we will attempt to develop a clearer picture of how digital consultations were initially introduced in different settings and how their use and implementation may have adapted and changed as the pandemic progressed. The second issue relates to the fact that not all of the evidence that we anticipate including in the review will come from the pandemic context. We will endeavour to include temporality as an aspect of our analysis for all the evidence by explicitly considering the stage of implementation of an innovation that is reported and by considering data related to change and adaptation over time (where reported). We plan to draw upon concepts from the Dynamic Sustainability Framework[78] to help 'sensitise' the team to issues of temporality. The framework focuses attention to the constantly changing evidence-base, the multi-level context in which interventions are delivered, and the broader ecological system within which the maternity care exists and operates.

For peer review only - http://bmjopen.bmj.com/site/about/guidelines.xhtml

The output of Phase 2 will be a comprehensive set of evidence tables and refined programme theories linked to associated sets of working papers organised by DC-CON type and purpose in relation to different points in the maternity care pathway.

Phase 3: Test and Refine Programme Theories (Validation)

The purpose of this Phase is to test and further refine the programme theories. This is done in three interlinked ways.

First, as part of an ongoing recursive approach, new literature searches will be undertaken to find, test and empirically explore any new theories identified during the review process.[67] This may also include a need to re-visit and re-code previously identified papers. As noted by Booth et al,[67] this stage may involve searches for specific named theories that have been identified in Phase 2 and that are considered worthy of further empirical exploration.

Second, further in-depth consultation/workshops with stakeholders will be undertaken to test and refine proposed theories. Our stakeholder groups will be asked to consider the proposed theories in relation to their own experiences, paying particular attention to clarifying the key mechanisms that are required to produce desired outcomes and to identify the key links between these mechanisms and different contexts. This process will help to identify 'gaps' that remain in our understanding and to clarify the essential elements of intervention and context that need to be place to ensure that the appropriate mechanisms can be activated.

Third, as described above, we will undertake a limited number of key informant interviews to test aspects of the programme theories and to evaluate their plausibility, credibility and transferability.[52]

The output of this Phase will be a theoretically-grounded explanatory framework for safe, appropriate and acceptable DC-CON in maternity care that can be used to guide intervention development, policy and practice.

Phase 4: Development of Actionable Recommendations

The purpose of this Phase is to generate actionable recommendations from the review. We will do this by holding an on-line webinar to which a much larger group of stakeholders will be invited. The purpose of this webinar will be to share and 'sense-check' the review findings and to solicit suggestions for recommendations, actions and strategies for dissemination. In addition, we will hold further meetings to discuss, develop and finalise recommendations appropriate to a range of constituents (researchers, commissioners, service leads, professionals, women, community organisations) and feasible for implementation in a UK NHS context.

ETHICS AND DISSEMINATION

For peer review only - http://bmjopen.bmj.com/site/about/guidelines.xhtml

Ethical approval for the key informant interviews has been obtained from the University of Nottingham Faculty of Medicine and Health Sciences Research Ethics Committee – reference no. FMHS 426-1221.

Dissemination strategies will be informed through ongoing engagement with our stakeholder groups. Key strategies will include academic papers, conference presentations, development of an online educational resource for health professionals and short papers in 'practice focused' journals.

AUTHOR'S CONTRIBUTIONS

The original protocol was developed with extensive input from all authors. CE prepared the protocol as a manuscript for publication. All authors have read, commented on and approved the final manuscript.

FUNDING STATEMENT

This work is supported by the UK National Institute for Health Research (NIHR), Health Services Delivery Research Programme, grant number: NIHR134535.

DISCLAIMER

The views expressed are those of the authors and not necessarily those of the NIHR or the UK Department of Health and Social Care.

COMPETING INTERESTS STATEMENT

None to declare

WORD COUNT

4,612

For peer review only - http://bmjopen.bmj.com/site/about/guidelines.xhtml

REFERENCES

1. National Maternity Review. Better Births: Improving Outcomes of Maternity Services in England: A Five Year Forward View for Maternity Care. 2016. Available from: https://www.england.nhs.uk/wp-content/uploads/2016/02/national-maternity-review-report.pdf (accessed 09/02/21)

2. NHS England. Maternity Transformation Programme. Available from: https://www.england.nhs.uk/mat-transformation/ (accessed 09/02/21)

3. NHS England. Better Births Four Years On: A Review of Progress 2020. Available from: https://www.england.nhs.uk/wp-content/uploads/2020/03/better-births-four-years-on-progress-report.pdf (accessed 09/02/21)

4. NHS Digital. Digital Maternity: Harnessing Digital Technology in Maternity Services. Contract No.: 09/02/21. Available from: https://digital.nhs.uk/services/digital-maternity-programme (accessed 25/01/22)

5. NHS Digital. Maternity DMA Report: Digital Maturity Assessment of Maternity Services in England. 2018. Available from: https://www.england.nhs.uk/wp-content/uploads/2018/11/national-maternity-dma-report.pdf (accessed 11/03/21)

6. Renfrew MJ, Cheyne H, Craig J, *et al.* Sustaining quality midwifery care in a pandemic and beyond. *Midwifery*. 2020;**88**:102759-59. doi:10.1016/j.midw.2020.102759

7. Jardine J, Relph S, Magee LA, *et al.* Maternity services in the UK during the coronavirus disease 2019 pandemic: a national survey of modifications to standard care. *BJOG*. 2020. doi:10.1111/1471-0528.16547

8. Fernandez Turienzo C, Newburn M, Agyepong A, *et al.* Addressing inequities in maternal health among women living in communities of social disadvantage and ethnic diversity. *BMC Public Health*. 2021;**21**:176. doi:10.1186/s12889-021-10182-4

9. Hinton L, Kuberska K, Dakin F, *et al.* Creating equitable remote antenatal care: the importance of inclusion. *BMJ Opinion*. 2021.

10. Royal College of Midwives. Virtual consultations: guidance on appropriate application for virtual consultations and practical tips for effective use (V2.3). 2020. Available from: https://www.rcm.org.uk/media/4596/virtual-consultations-v23-revised-17-december-2021-review-25-january-2021.pdf (accessed 05/02/21)

11. Royal College of Midwives, Royal College of Obstetricians & Gynaecologists. Guidance for Antenatal and Postnatal Services in the evolving Coronavirus (COVID-19) pandemic (Version 3). 2020. Available from: https://www.rcog.org.uk/globalassets/documents/guidelines/2020-10-21-guidance-for-antenatal-and-postnatal-services-in-the-evolving-coronavirus-covid-19-pandemic-v3.pdf (accessed 05/02/21)

12. Saad M, Chan S, Nguyen L, *et al.* Patient perceptions of the benefits and barriers of virtual postnatal care: a qualitative study. *BMC Pregnancy and Childbirth*. 2021;**21**:543-43. doi:10.1186/s12884-021-03999-9

For peer review only - http://bmjopen.bmj.com/site/about/guidelines.xhtml

13. Riley V, Ellis N, Mackay L, *et al.* The impact of COVID-19 restrictions on women's pregnancy and postpartum experience in England: A qualitative exploration. *Midwifery*. 2021;**101**:103061-61. doi:10.1016/j.midw.2021.103061

14. John JR, Curry G, Cunningham-Burley S. Exploring ethnic minority women's experiences of maternity care during the SARS-CoV-2 pandemic: a qualitative study. *BMJ Open*. 2021;**11**:e050666. doi:10.1136/bmjopen-2021-050666

15. McKinlay AR, Fancourt D, Burton A. Factors affecting the mental health of pregnant women using UK maternity services during the COVID-19 pandemic: A qualitative interview study. *medRxiv*. 2021:2021.10.20.21265279. doi:10.1101/2021.10.20.21265279

16. Tavener CR, Kyriacou C, Elmascri I, *et al.* Rapid introduction of virtual consultation in a hospital-based Consultant-led Antenatal Clinic to minimise exposure of pregnant women to COVID-19. *BMJ Open Quality*. 2022;**11**:e001622. doi:10.1136/bmjoq-2021-001622

17. Brislane Á, Larkin F, Jones H, *et al.* Access to and Quality of Healthcare for Pregnant and Postpartum Women During the COVID-19 Pandemic. *Frontiers in Global Women's Health*. 2021;**2**. doi:10.3389/fgwh.2021.628625

18. Quinn LM, Olajide O, Green M, *et al.* Patient and Professional Experiences With Virtual Antenatal Clinics During the COVID-19 Pandemic in a UK Tertiary Obstetric Hospital: Questionnaire Study. *J Med Internet Res*. 2021;**23**:e25549. doi:10.2196/25549

19. Sanders J, Blaylock R, WRISK Study Team. Users' experiences of COVID-19 maternity service changes (Version 2). 2020. Available from: https://wrisk.org/wp-content/uploads/2021/02/WRISK-COVID-Report_Dec20_V2.pdf (accessed 05/02/21)

20. Gülmezoglu AM, Ammerdorffer A, Narasimhan M, *et al.* Self-care and remote care during pregnancy: a new paradigm? *Health Research Policy and Systems*. 2020;**18**:107. doi:10.1186/s12961-020-00627-4

21. Royal College of Midwives. Digital Technology in Maternity Care: A Position Statement 2021. Available from: https://www.rcm.org.uk/media/4789/rcm_position-statement_digital-technologies_final.pdf (accessed 16/03/21)

22. The Health Foundation. Securing a Positive Healthcare Technology Legacy from Covid-19. 2021. Available from: https://www.health.org.uk/publications/long-reads/securing-a-positive-health-care-technology-legacy-from-covid-19 (accessed 08-04-21)

23. Bick D, Cheyne H, Chang Y-S, *et al.* Maternal postnatal health during the COVID-19 pandemic: Vigilance is needed. *Midwifery*. 2020;**88**:102781. doi:https://doi.org/10.1016/j.midw.2020.102781

24. NHS England & NHS Improvement. Equity and equality: Guidance for local maternity systems. 2021. Available from: https://www.england.nhs.uk/wp-content/uploads/2021/09/C0734-equity-and-equality-guidance-for-local-maternity-systems.pdf (accessed 07/02/22)

25. World Health Organisation. Classification of Digital Health Interventions v1.0. 2017. Available from: https://apps.who.int/iris/bitstream/handle/10665/260480/WHO-RHR-18.06-eng.pdf;jsessionid=1DA9EAD9506E29BB5E1ED23FB797EF9F?sequence=1 (accessed 16/03/21)

For peer review only

For peer review only - http://bmjopen.bmj.com/site/about/guidelines.xhtml

26. Griffiths FE, Armoiry X, Atherton H, *et al.* The role of digital communication in patient-clinician communication for NHS providers of specialist clinical services for young people [the Long-term conditions Young people Networked Communication (LYNC) study]: a mixed-methods study. *Health Services and Delivery Research*. 2018;**6**. doi:https://doi.org/10.3310/hsdr06090

27. Lee Y, Cho S. Technology-Supported Interventions for Pregnant Women: A Systematic Review. *Comput Inform Nurs*. 2019;**37**:501-12. doi:10.1097/cin.0000000000000535

28. van den Heuvel JF, Groenhof TK, Veerbeek JH, *et al.* eHealth as the Next-Generation Perinatal Care: An Overview of the Literature. *J Med Internet Res*. 2018;**20**:e202. doi:10.2196/jmir.9262

29. DeNicola N, Grossman D, Marko K, *et al.* Telehealth Interventions to Improve Obstetric and Gynecologic Health Outcomes: A Systematic Review. *Obstet Gynecol*. 2020;**135**:371-82. doi:10.1097/aog.0000000000003646

30. Lavender T, Richens Y, Milan SJ, *et al.* Telephone support for women during pregnancy and the first six weeks postpartum. *Cochrane Database of Systematic Reviews*. 2013. doi:10.1002/14651858.CD009338.pub2

31. Kapinos K, Kotzias V, Bogen D, *et al.* The Use of and Experiences With Telelactation Among Rural Breastfeeding Mothers: Secondary Analysis of a Randomized Controlled Trial. *J Med Internet Res*. 2019;**21**:e13967. doi:10.2196/13967

32. Habibi MF, Nicklas J, Spence M, *et al.* Remote lactation consultation: a qualitative study of maternal response to experience and recommendations for survey development. *J Hum Lact*. 2012;**28**:211-7. doi:10.1177/0890334411432716

33. Ames HMR, Glenton C, Lewin S, *et al.* Clients' perceptions and experiences of targeted digital communication accessible via mobile devices for reproductive, maternal, newborn, child, and adolescent health: a qualitative evidence synthesis. *Cochrane Database of Systematic Reviews*. 2019. doi:10.1002/14651858.CD013447

34. Bailey CM, Newton JM, Hall HG. Telephone triage and midwifery: A scoping review. *Women Birth*. 2018;**31**:414-21. doi:10.1016/j.wombi.2017.12.002

35. Jeganathan S, Prasannan L, Blitz MJ, *et al.* Adherence and acceptability of telehealth appointments for high-risk obstetrical patients during the coronavirus disease 2019 pandemic. *Am J Obstet Gynecol MFM*. 2020;**2**:100233. doi:10.1016/j.ajogmf.2020.100233

36. Khalil C. Understanding the Adoption and Diffusion of a Telemonitoring Solution in Gestational Diabetes Mellitus: Qualitative Study. *JMIR Diabetes*. 2019;**4**:e13661. doi:10.2196/13661

37. Butler Tobah YS, LeBlanc A, Branda ME, *et al.* Randomized comparison of a reduced-visit prenatal care model enhanced with remote monitoring. *Am J Obstet Gynecol*. 2019;**221**:638.e1-38.e8. doi:10.1016/j.ajog.2019.06.034

38. Marko KI, Ganju N, Brown J, *et al.* Remote Prenatal Care Monitoring With Digital Health Tools Can Reduce Visit Frequency While Improving Satisfaction. *Obstetrics & Gynecology*. 2016;**127**:1S.

For peer review only - http://bmjopen.bmj.com/site/about/guidelines.xhtml

39. Panda S, O'Malley D, Barry P, *et al.* Women's views and experiences of maternity care during COVID-19 in Ireland: A qualitative descriptive study. *Midwifery*. 2021;**103**:103092. doi:https://doi.org/10.1016/j.midw.2021.103092

40. Seguranyes G, Costa D, Fuentelsaz-Gallego C, *et al.* Efficacy of a videoconferencing intervention compared with standard postnatal care at primary care health centres in Catalonia. *Midwifery*. 2014;**30**:764-71. doi:https://doi.org/10.1016/j.midw.2013.08.004

41. Feroz A, Perveen S, Aftab W. Role of mHealth applications for improving antenatal and postnatal care in low and middle income countries: a systematic review. *BMC Health Services Research*. 2017;**17**:704. doi:10.1186/s12913-017-2664-7

42. Linden K, Domgren N, Zaigham M, *et al.* Being in the shadow of the unknown — Swedish women's lived experiences of pregnancy during the COVID-19 pandemic, a phenomenological study. *Women and Birth*. 2021. doi:https://doi.org/10.1016/j.wombi.2021.09.007

43. Karavadra B, Stockl A, Prosser-Snelling E, *et al.* Women's perceptions of COVID-19 and their healthcare experiences: a qualitative thematic analysis of a national survey of pregnant women in the United Kingdom. *BMC Pregnancy and Childbirth*. 2020;**20**:600. doi:10.1186/s12884-020-03283-2

44. Penny RA, Bradford NK, Langbecker D. Registered nurse and midwife experiences of using videoconferencing in practice: A systematic review of qualitative studies. *J Clin Nurs*. 2018;**27**:e739-e52. doi:10.1111/jocn.14175

45. Harrison TN, Sacks DA, Parry C, *et al.* Acceptability of Virtual Prenatal Visits for Women with Gestational Diabetes. *Women's Health Issues*. 2017;**27**:351-55. doi:https://doi.org/10.1016/j.whi.2016.12.009

46. Liu CH, Goyal D, Mittal L, *et al.* Patient Satisfaction with Virtual-Based Prenatal Care: Implications after the COVID-19 Pandemic. *Matern Child Health J*. 2021;**25**:1735-43. doi:10.1007/s10995-021-03211-6

47. Kolker S, Biringer A, Bytautas J, *et al.* Pregnant during the COVID-19 pandemic: an exploration of patients' lived experiences. *BMC Pregnancy and Childbirth*. 2021;**21**:851-51. doi:10.1186/s12884-021-04337-9

48. Atmuri K, Sarkar M, Obudu E, *et al.* Perspectives of pregnant women during the COVID-19 pandemic: A qualitative study. *Women and Birth*. 2021. doi:https://doi.org/10.1016/j.wombi.2021.03.008

49. Keating N, Dempsey B, Corcoran S, *et al.* Women's Experience of Pregnancy and Birth during the Covid-19 Pandemic: A Qualitative Study. Research Square; 2020.

50. Altman MR, Gavin AR, Eagen-Torkko MK, *et al.* Where the System Failed: The COVID-19 Pandemic's Impact on Pregnancy and Birth Care. *Global Qualitative Nursing Research*. 2021;**8**:23333936211006397. doi:10.1177/23333936211006397

51. Fryer K, Delgado A, Foti T, *et al.* Implementation of Obstetric Telehealth During COVID-19 and Beyond. *Matern Child Health J*. 2020;**24**:1104-10. doi:10.1007/s10995-020-02967-7

For peer review only - http://bmjopen.bmj.com/site/about/guidelines.xhtml

52. Pawson R, Greenhalgh T, Harvey G, *et al.* Realist review--a new method of systematic review designed for complex policy interventions. *J Health Serv Res Policy*. 2005;**10 Suppl 1**:21-34. doi:10.1258/1355819054308530

53. Pawson R. Evidence Based Policy: A Realist Perspective. London: Sage; 2006.

54. Bhaskar R. A Realist Theory of Science. Leeds: Leeds Books; 2008.

55. Archer M. Realist Social Theory: The Morphogenic Approach. Cambridge: Cambridge University Press; 1995.

56. Emmel N, Manzano A, Dalkin S, *et al.* Doing Realist Research. London: Sage 2018.

57. Bauer MS, Damschroder L, Hagedorn H, *et al.* An introduction to implementation science for the non-specialist. *BMC Psychology*. 2015;**3**:32. doi:10.1186/s40359-015-0089-9

58. Craig P, Dieppe P, Macintyre S, *et al.* Developing and evaluating complex interventions: the new Medical Research Council guidance. *BMJ*. 2008;**337**:a1655. doi:10.1136/bmj.a1655

59. Pawson R, Tilley N. Realistic Evaluation. London: Sage; 1997.

60. Rycroft-Malone J, McCormack B, Hutchinson AM, *et al.* Realist synthesis: illustrating the method for implementation research. *Implementation Science*. 2012;**7**:33. doi:10.1186/1748-5908-7-33

61. Dalkin SM, Greenhalgh J, Jones D, *et al.* What's in a mechanism? Development of a key concept in realist evaluation. *Implementation Science*. 2015;**10**:49. doi:10.1186/s13012-015-0237-x

62. Shaw J, Gray CS, Baker GR, *et al.* Mechanisms, contexts and points of contention: operationalizing realist-informed research for complex health interventions. *BMC Medical Research Methodology*. 2018;**18**:178. doi:10.1186/s12874-018-0641-4

63. Wong G, Westthorp G, Pawson R, *et al.* Realist Synthesis: RAMESES Training Materials. 2013. Available from: https://www.ramesesproject.org/media/Realist_reviews_training_materials.pdf (accessed 02-02-21)

64. Hunter R, Gorely T, Beattie M, *et al.* Realist review. *International Review of Sport and Exercise Psychology*. 2022:1-24. doi:10.1080/1750984X.2021.1969674

65. Wong G, Greenhalgh T, Westhorp G, *et al.* RAMESES publication standards: realist syntheses. *BMC Medicine*. 2013;**11**:21. doi:10.1186/1741-7015-11-21

66. Evans K, Janiszewski H, Evans C, *et al.* Establishing information needs and research priorities in response to the Covid-19 pandemic in the local maternity setting. *Midwifery*. 2021;**95**:102922. doi:10.1016/j.midw.2021.102922

67. Booth A, Wright J, Briscoe S. Scoping and searching to support realist approaches In: Emmel N, Greenhalgh J, Manzano A*et al*, editors. Doing Realist Research. London: Sage Publications; 2018. p. 147-66. (accessed

68. Booth A, Briscoe S, Wright JM. The "realist search": A systematic scoping review of current practice and reporting. *Res Synth Methods*. 2020;**11**:14-35. doi:10.1002/jrsm.1386

For peer review only - http://bmjopen.bmj.com/site/about/guidelines.xhtml

69. Gale NK, Heath G, Cameron E, *et al.* Using the framework method for the analysis of qualitative data in multi-disciplinary health research. *BMC Medical Research Methodology*. 2013;**13**:117. doi:10.1186/1471-2288-13-117

70. Greenhalgh T, Shaw S, Wherton J, *et al.* Real-World Implementation of Video Outpatient Consultations at Macro, Meso, and Micro Levels: Mixed-Method Study. *Journal of Medical Internet Research*. 2018;**20**:e150-e50. doi:10.2196/jmir.9897

71. Vassilev I, Rowsell A, Pope C, *et al.* Assessing the implementability of telehealth interventions for self-management support: a realist review. *Implement Sci*. 2015;**10**:59. doi:10.1186/s13012-015-0238-9

72. May C, Finch T, Mair F, *et al.* Understanding the implementation of complex interventions in health care: the normalization process model. *BMC Health Serv Res*. 2007;**7**:148. doi:10.1186/1472-6963-7-148

73. Booth A, Harris J, Croot E, *et al.* Towards a methodology for cluster searching to provide conceptual and contextual "richness" for systematic reviews of complex interventions: case study (CLUSTER). *BMC Med Res Methodol*. 2013;**13**:118. doi:10.1186/1471-2288-13-118

74. Williams L, Rycroft-Malone J, Burton CR, *et al.* Improving skills and care standards in the support workforce for older people: a realist synthesis of workforce development interventions. *BMJ Open*. 2016;**6**:e011964. doi:10.1136/bmjopen-2016-011964

75. Popay J, Rogers A, Williams G. Rationale and standards for the systematic review of qualitative literature in health services research. *Qual Health Res*. 1998;**8**:341-51. doi:10.1177/104973239800800305

76. Pawson R. Digging for nuggets: how 'bad' research can yield 'good' evidence. *International Journal of Social Research Methodology*. 2006;**9**:127-42.

77. May C. Towards a general theory of implementation. *Implementation Science*. 2013;**8**:18. doi:10.1186/1748-5908-8-18

78. Chambers DA, Glasgow RE, Stange KC. The dynamic sustainability framework: addressing the paradox of sustainment amid ongoing change. *Implementation Science*. 2013;**8**:117. doi:10.1186/1748-5908-8-117

For peer review only

Supplemental File 1: Digital Clinical Consultation in Maternity Care: Realist Review Protocol Process Flowchart

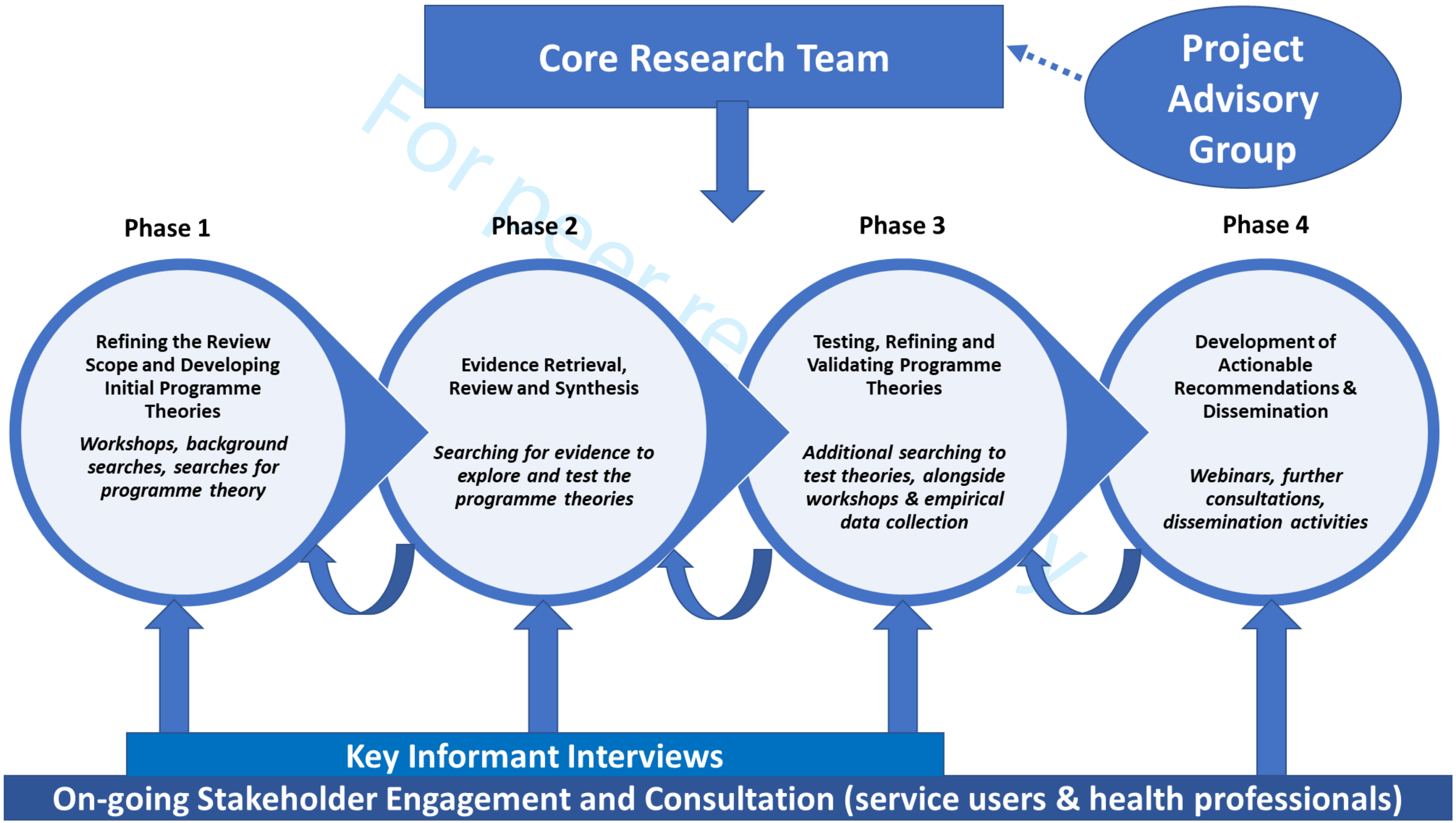

Supplemental File No. 2: Initial Search Strategy

Database: Ovid MEDLINE(R) and Epub Ahead of Print, In-Process, In-Data-Review & Other Non-Indexed Citations, Daily and Versions(R) <1946 to January 27, 2022>

#	Query	Results from 28 Jan 2022
1	exp Telemedicine/	38,848
2	remote consultation/ or videoconferencing/	7,285
3	(telemedicine or tele-medicine or telecare or tele-care or telehealth or tele-health).mp. [mp=title, abstract, original title, name of substance word, subject heading word, floating sub-heading word, keyword heading word, organism supplementary concept word, protocol supplementary concept word, rare disease supplementary concept word, unique identifier, synonyms]	44,080
4	((remote* or virtual* or online or on-line or digital*) adj3 (consultation* or appointment* or meet*)).mp.	8,514
5	(videoconferenc* or video-conferenc* or teleconferenc* or tele-conferenc* or zoom or facetime or face-time).mp.	8,842
6	1 or 2 or 3 or 4 or 5	56,928
7	exp Maternal Health Services/	54,815
8	exp Prenatal Care/ or exp Midwifery/ or exp Pregnancy/ or exp Obstetrics/	972,055
9	(matern* or pregnan* or prenatal or pre-natal or antenatal or ante-natal or perinatal or peri-natal or postnatal or post-natal or postpartum or post-partum or breastfeed* or breast feed* or midwi* or obstetric*).mp.	1,408,667
10	7 or 8 or 9	1,420,532
11	6 and 10	1,835
12	Primary Health Care/	86,523
13	general practice/ or family practice/	77,081
14	(primary care or general practice or GP or family doctor* or family physician*).mp.	225,104
15	exp Endocrinologists/	222
16	exp Cardiologists/	986
17	(endocrinologist* or cardiologist*).mp.	21,716
18	12 or 13 or 14 or 15 or 16 or 17	309,535
19	6 and 18	4,484
20	19 not 11	4,360
21	(framework* or model* or theor* or concept*).mp.	5,371,555
22	limit 21 to yr="2010 -Current"	3,213,643
23	11 and 22	407
24	20 and 22	1,060

For peer review only

BMJ Open

A Realist Inquiry into Maternity Care @ a Distance (ARM@DA): Realist Review Protocol

Journal:	*BMJ Open*
Manuscript ID	bmjopen-2022-062106.R1
Article Type:	Protocol
Date Submitted by the Author:	21-Jul-2022

Complete List of Authors:	Evans, Catrin; University of Nottingham, School of Health Sciences,<br>Evans , Kerry; University of Nottingham, School of Health Sciences,<br>Booth, Andrew; The University of Sheffield, ScHARR<br>Timmons, Stephen; University of Nottingham, Business School<br>Jones, Nia; University of Nottingham, School of Medicine<br>Nazmeen, Benash; University of Nottingham, School of Health Sciences,<br>Sunney, Candice ; Nottingham Maternity Research Network<br>Clowes, Mark; The University of Sheffield, ScHARR<br>Clancy, Georgia; University of Nottingham, School of Health Sciences,<br>Spiby, Helen; University of Nottingham, School of Health Sciences

**Primary Subject Heading**:	Health services research
Secondary Subject Heading:	Evidence based practice, Obstetrics and gynaecology, Communication
Keywords:	Organisation of health services < HEALTH SERVICES ADMINISTRATION & MANAGEMENT, Maternal medicine < OBSTETRICS, QUALITATIVE RESEARCH, STATISTICS & RESEARCH METHODS, Telemedicine < BIOTECHNOLOGY & BIOINFORMATICS

SCHOLARONE™
Manuscripts

A Realist Inquiry into Maternity Care @ a Distance (ARM@DA): Realist Review Protocol

- Catrin Evans [*Corresponding Author*], School of Health Sciences, University of Nottingham, Nottingham, U.K. NG7 2UH, catrin.evans@nottingham.ac.uk, Tel. +44 115 823 0894; ORCID: 0000-0002-5338-2191
- Kerry Evans, School of Health Sciences, University of Nottingham, Nottingham, UK
- Andrew Booth, School of Health and Related Research, University of Sheffield, Sheffield, UK
- Stephen Timmons, Business School, University of Nottingham, Nottingham, UK
- Nia Jones, School of Medicine, University of Nottingham, Nottingham, UK
- Benash Nazmeen, School of Health Sciences, University of Nottingham, Nottingham, UK
- Candice Sunney, Nottingham Maternity Research Network, Nottingham, UK
- Mark Clowes, School of Health and Related Research, University of Sheffield, Sheffield, UK
- Georgia Clancy, School of Health Sciences, University of Nottingham, Nottingham, UK
- Helen Spiby, School of Health Sciences, University of Nottingham, Nottingham, UK

Keywords

- realist review; remote care; digital clinical consultation; virtual consultation; tele-health;

Word Count

5,490

For peer review only

ABSTRACT

INTRODUCTION

One of the most commonly reported Covid-19-related changes to all maternity services has been an increase in the use of digital clinical consultations such as telephone or video-calling, however the ways in which they can be optimally utilised along maternity care pathways remains unclear. It is imperative that digital service innovations do not further exacerbate (and, ideally, should tackle) existing inequalities in service access and clinical outcomes. Using a realist approach, this project aims to synthesise the evidence around implementation of digital clinical consultations, seeking to illuminate how they can work to support safe, personalised and appropriate maternity care and to clarify when they might be most appropriately used, for whom, when, and in what contexts?

METHODS AND ANALYSIS

The review will be conducted in 4 iterative phases, with embedded stakeholder involvement: (i) refining the review focus and generating initial programme theories, (ii) exploring and developing the programme theories in light of evidence, (iii) testing/refining the programme theories, and, (iv) constructing actionable recommendations. The review will draw upon four sources of evidence: (i) published literature (searching 9 bibliographic databases), (ii) unpublished (grey) literature, including research, audit, evaluation and policy documents (derived from Google Scholar, website searches and e-thesis databases), (iii) expertise contributed by service user and health professional stakeholder groups (n=20-35), and (iv) key informant interviews (n=12). Included papers will consist of any study design, in English and from 2010 onwards. The review will follow the RAMESES (Realist and Meta-narrative Evidence Synthesis Evolving Standards) quality procedures and reporting guidance.

ETHICS AND DISSEMINATION

Ethical approval has been obtained from the University of Nottingham, Faculty of Medicine and Health Sciences Ethics Committee (FMHS 426-1221). Informed consent will be obtained for all key informant interviews. Findings will be disseminated in a range of formats relevant to different audiences.

PROSPERO REGISTRATION

No. CRD42021288702

For peer review only - http://bmjopen.bmj.com/site/about/guidelines.xhtml

STRENGTHS AND LIMITATIONS OF THIS STUDY

- A realist synthesis moves beyond effectiveness and acceptability to consider evidence related to implementation (what works, for whom and under what contexts).
- Stakeholder engagement (service users and health professionals) is embedded at every stage of the review.
- The focus of a realist review on mechanisms underlying implementation will yield insights that are potentially transferable across geographical and clinical settings.
- Research on digital communication in healthcare is a fast evolving field, so it is possible that new evidence may be missed, although this may be mitigated to some extent by the iterative approach to searching within a realist review that will support the identification of relevant evidence, even after the initial searches have taken place.

For peer review only

For peer review only - http://bmjopen.bmj.com/site/about/guidelines.xhtml

INTRODUCTION

This article sets out a protocol for a realist review to generate a theory- and evidence-informed framework to guide best practice in the implementation of digital clinical consultations in maternity care. The project is being undertaken by a UK-based team and is oriented to producing findings that are actionable in the context of the UK's National Health Service (NHS). Nonetheless, the project will draw upon evidence from comparable geographical and health system settings and hence its findings will be of international relevance.

Maternity care in England is undergoing a substantive transformation programme whose overall aim is to support services to work across professional and service boundaries to become safer, kinder, more personalised, and more family-friendly.[1-3] A core work stream is 'harnessing digital technology'.[4, 5] In the UK, as in many other countries, the Covid-19 pandemic has radically altered and dramatically accelerated the context of this digital policy imperative.[6-9]

One of the most commonly reported Covid-related changes to all forms of service provision in maternity care has been an increase in the use of remote/virtual consultations using telephone or video.[4, 7, 10-18] For example, one recent survey of all 194 obstetric units in England (42% response rate) found that 89% reported using digitally delivered consultation methods in antenatal care and 56.8% for postnatal care.[7] For antenatal services, the majority of these (87.7%) were conducted via the telephone whereas a smaller percentage were conducted using everyday video technology or specialist video technology (12% and 25.9% respectively). A UK survey of 524 women in 2020 reported that 51.8% had experienced telephone or video consultations.[19]

Within maternity care, consultations have a wide range of potential purposes depending upon the individual circumstances and characteristics of the service user, clinical categorisation of the pregnancy (e.g. high risk or low risk) and stage of the maternity care pathway (e.g. antenatal care, assessment of early labour, postnatal care). Consultations may involve clinical (physical and mental health) assessment, monitoring, safeguarding assessments, health promotion, information giving, education or therapeutic support. The interactions need to be organised and implemented within multi-professional care pathways that seek to promote continuity, personalisation and choice, recognise diversity and ensure safety.[9] Likewise, consultations need to be supported by auditable records that can be accessed by, and strengthen communications between, different providers and settings.

The rapid shift to remote or digitally delivered clinical consultations was initially implemented to support pandemic response objectives such as social distancing and service demand management.[6, 9, 16, 20-22] Going forward however, a key challenge for maternity services lies in how to 're-purpose' the use of remote consultations to serve longer term quality, equity and productivity objectives.[6, 9] Although remote consultations may now be widespread, the ways in which they can optimally be utilised in future along maternity care pathways remains unclear. In particular, it is imperative that service innovations do not further exacerbate (and, ideally, should tackle) existing inequalities in service access and clinical outcomes.[9, 14, 23, 24]

For peer review only - http://bmjopen.bmj.com/site/about/guidelines.xhtml

Hence, there is a need for a comprehensive review of the evidence base to inform future service developments and future research.[6]

Operational Definitions

Digitally-facilitated clinical communication has a highly diverse nomenclature, including terms such as telehealth, telemedicine, virtual/remote care, video consultations and digital consultation (amongst many others). In spite of efforts by WHO in 2017 to standardise concepts and terminology, there is no internationally agreed or consistent set of terms, meanings or definitions.[25] A similar diversity is found in the rapidly evolving technologies and systems used to implement digitally-facilitated consultations. Key distinctions are that digital technologies can be utilised for: (i) direct 'live' clinical communication/consultation between a service user and a practitioner (synchronous), or (ii) direct communication that may happen at different time points (asynchronous), such as text messaging. This is in contrast to other digital care modalities such as remote monitoring systems (e.g. remote blood pressure monitoring), on-line triage algorithms, the use of apps, wearable personal devices or electronic medical records which are usually asynchronous and do not involve direct interpersonal patient/practitioner communication (although these may be utilised to trigger, or to directly inform, consultations).

The focus of this project is on maternity care **consultations** that are facilitated through, and/or complemented by, digital technology. To capture this focus, we will use the term 'Digital Clinical Consultation' – henceforth referred to as 'DC-CON'.[26] The working definition for our project is outlined in Table 1:

Table 1: Operational Definition

Digital Clinical Consultation (DC-CON) *– Synchronous telephone or video consultations involving direct interaction between a service user and a maternity healthcare professional. It has two-way functionality and can be initiated by either party. It may be linked to, or complemented by, other digital technologies within the maternity care pathways.*

This definition recognises that the focus is on the consultation itself, but that this might be linked to, or informed by, other digital technologies.

This project will investigate digital consultations across the maternity pathway, i.e. during pregnancy, the intrapartum period and early postnatal care (up to 10-14 days post-partum).

Existing Evidence on DC-CON in Maternity Care

There is a large and rapidly expanding literature associated with DC-CON in maternity care.[20] For simplicity, we conceptualise the purpose of DC-CON in two ways (while recognising significant overlap between the two). The first relates to DC-CON that is additional to usual care – where specialist support is required on a single issue where a specific need is identified (e.g. interventions to support perinatal mental health, breastfeeding or smoking cessation). There is now substantial evidence that such targeted interventions can be feasible, acceptable and effective to varying degrees.[20, 27-33]

For peer review only - http://bmjopen.bmj.com/site/about/guidelines.xhtml

The second DC-CON purpose relates to situations where DC-CON is already the main modality of care (e.g. in telephone helplines or triage systems).[34] It also refers to situations where standard care pathways are altered so that some in-person points of contact are replaced or supplemented by DC-CON (as happened during the Covid-19 pandemic). DC-CON has been investigated with regard to antenatal care among both high[35, 36] and low[37, 38] risk women. For example, a recent randomised control trial in the USA sought to compare standard in-person antenatal care with a new 'hybrid' system that included virtual consultations. This study found higher satisfaction with care, no difference in health outcomes and less anxiety in the hybrid care group.[37] However, a systematic review of telephone support for women in pregnancy and postpartum was less conclusive, with results suggesting that telephone support may be associated with higher overall satisfaction with care while yielding uncertain impacts on clinical outcomes.[30]

Overall, existing research on DC-CON in maternity care shows it can be safe and acceptable in controlled conditions.[27-29, 35, 38-40 37, 41] Research also shows that the experiences of staff and women with DC-CON vary quite significantly.[42 7, 12, 34, 39, 43-50] A gap remains therefore in understanding the conditions required for safe and acceptable DC-CON implementation and in understanding the factors that underpin variation in experience. For example: variations between women of different ethnicities (to ensure that DC-CON does not further exacerbate racial inequalities in maternity care);[51] how women who face communication barriers will be supported by workers to understand their care and make choices via DC-CON;[16, 52-55] and how access to the technologies necessary for DC-CON will vary between services users and service providers as a result of financial resources, internet connectivity, digital literacy and the digital maturity of NHS services.[4, 52, 56] Such variation is problematic, as it is implementation in real-world contexts, at scale, that needs to be understood for the systems-wide transformation as envisaged in the NHS Maternity Transformation Programme.[2, 21, 57] It is this gap that the proposed project will address.

Research Aim and Question

This project aims to undertake an in-depth and theory informed investigation of the evidence around implementation of digital clinical consultations. It seeks to illuminate how digital consultations can work to support safe, personalised and appropriate maternity care and to clarify when they might be most appropriately used, for whom, when, and in what contexts.

Scoping searches of PROSPERO, Medline and the Cochrane Library have not identified any similar reviews currently being undertaken.

PHILOSOPHY AND METHODOLOGY

We will undertake a realist synthesis in which patient and public involvement (PPI) and diverse stakeholder participation is embedded at every stage.[58] Realist syntheses seek to investigate the relationships between what can be observed and experienced, human interpretation of this reality and 'unseen' underlying social structures.[59-61] Increasingly, realist approaches are applied in health research to investigate the complexity of health-related behaviour and to explore how behaviours are shaped by human agency via intersecting social structures that manifest differently in different contexts.[62] Realist approaches are particularly well suited to investigating complex interventions such as DC-CON.[63, 64] This is

For peer review only - http://bmjopen.bmj.com/site/about/guidelines.xhtml

because realist inquiry seeks to establish causal relationships - expressed as 'programme theories' - between intersecting intervention components, contexts and outcomes.[65] When applied to evidence synthesis, a realist approach focuses on understanding *how* particular interventions lead to particular outcomes under particular contexts (i.e. 'how does A lead to B'? and 'how might B be affected when A is implemented through contexts C, D or E'?).[66] This contrasts with the linear or deterministic approaches adopted by conventional systematic reviews which ask: 'does A lead to B'? (e.g. an effectiveness review) or: 'is A acceptable or meaningful to a particular group of individuals in a particular context'? (e.g. a qualitative review).[58]

As applied to our research question, the logic of a realist review proposes that different types of interventions (e.g. a video call or a phone call) supply particular resources into a situation that prompt diverse possible reactions and responses (also referred to as 'reasonings') from women and health professionals.[67] The interaction between the resource and the response constitutes a 'mechanism.'[67] Mechanisms are influenced by differences in how an intervention is delivered but also by the context of particular women's lives or different service configurations.[67] This means that the acceptability and outcomes of DC-CON may be highly contextually contingent.[68] A realist approach seeks to identify certain patterns ('demi-regularities'[59]) between types of resource (e.g. a phone call), how individuals respond and how a particular context may alter these responses. Hence, a realist review is focused on identifying and testing programme theories that can account for the contingent nature of intervention implementation.[69] These mid-range explanatory theories are a principal output from a realist review. Their insights make a substantive contribution to health service innovation because, by taking context into account, they are potentially 'transferable' – producing insights relevant to a wide range of settings and geographical contexts.[70]

Our proposed realist synthesis will be conducted in 4 iterative phases (see below). The review will follow the RAMESES (Realist and Meta-narrative Evidence Synthesis Evolving Standards) quality procedures[69] and, subsequently, will comply with RAMESES reporting guidance.[71]

Each phase of the review potentially draws upon three sources of evidence: (i) literature (published and unpublished research, audit, evaluation and theory), (ii) diverse stakeholder expertise and insights, and (iii) key informant interviews.

METHODS AND ANALYSIS

Patient and Public Involvement

This project has been informed by service user involvement from its outset. and seeks to follow the NIHR UK Standards for Public Involvement in Research.[72] Public involvement in the project will be reported following the GRIPP2 reporting checklist.[73]

A maternity research priority setting exercise was undertaken in 2020 by some members of the research team in which a PPI research network played a key role (the Nottingham Maternity Research Network - NMRN).[74] DC-CON was identified as an important topic for future work. A member of the NMRN (CS) subsequently became a co-investigator on the current project. The NMRN has been instrumental in shaping the current project. For

For peer review only - http://bmjopen.bmj.com/site/about/guidelines.xhtml

example, they identified a need to increase the diversity of PPI within the project. As a result, two other organisations were invited to become involved: (i) Women, Health and Family Services (based in east London serving deprived communities in an ethnically and linguistically diverse area and which runs an award winning ‘Maternity Mates’ community volunteer service), and (ii) the National Autistic Society (this has national representation and has been undertaking several recent projects on neuro-diversity and pregnancy/motherhood). Within the project structure, these 2 organisations have been grouped together with the NMRN to form a ‘Community Organisation and Service User Stakeholder Group’ (COSU-SG). The COSU-SG enables a wide spectrum of socio-economic, geographical, ethnic and neuro-diversity amongst women to be represented. Each of the 3 organisations has a named lead for involvement in the project. Up to 5 individuals from each organisation will attend project stakeholder events (the organisational lead and 4 others). The organisational leads are responsible for selecting the most appropriate individuals from their organisations to engage with project events and will provide them with ongoing support and information. They will also facilitate consultation and engagement with their wider networks where required (e.g. through social media or email lists or by suggesting particular individuals as key informants).

In addition to the COSU-SG, all project phases will also be informed by a health professional stakeholder group (HP-SG). The HP-SG will include between 10-20 obstetricians, digital midwives, frontline and managerial midwifery staff from different maternity settings – recruited via social media and through professional networks. Group membership is not bounded and fixed, however, but may evolve as the project progresses to ensure that appropriate expertise and insights are accessed. The purpose of the two stakeholder/PPI groups is to shape, participate in, contribute to, and advise the research team on all project phases to ensure that project questions, programme theories and recommendations reflect diverse real-world experiences and are relevant.

For both groups of stakeholders, anyone expressing an interest to get involved will first be contacted by the project PI (CE) for an in-depth discussion about the project and their potential contribution and commitment. They will also be sent written information about the study (this information sheet will be reviewed for readability/plain English). It will be stressed that their involvement is entirely voluntary and that they can withdraw at any time. Subsequently, the PPI co-applicant (CS) will contact individuals periodically to offer an opportunity for further discussion, to ask questions and to ensure that they remain happy to contribute. All personal details about stakeholders and records of project meetings/workshops will be securely stored in password protected files according to a University-approved Data Management Plan.

Overarching Approach to Literature Searching

Within this realist review, the overall search approach will follow the only published systematic approach to the “realist search”.[75, 76] This approach extends and enhances the Task and Time Template for a realist review advanced by Pawson.[59] It outlines 4 separate and distinct phases of searching using different retrieval techniques and targeted at different evidence bases, “topped” and “tailed” by precise question formulation and meticulous documentation.[75]

For peer review only - http://bmjopen.bmj.com/site/about/guidelines.xhtml

The search strategies within each phase will be developed and operationalised by experienced information specialists (AB & MC). The search dates will be restricted to evidence from 2010 to the present to reflect the need for contemporary data. Likewise, inclusion of evidence will be limited to reports and studies in the English language and in high-income settings (OECD countries). The search approach will engage the project advisory group and stakeholders throughout, consulting them where required to provide advice (for example clarifying the terminology used at service level) and help with identifying relevant literature (including, for example, cascading requests to identify literature and policy/practice documents through their associated social media sites and websites).

Key Informant Interviews

Data sources for the project may also involve a small number of key informant interviews. Within a realist review, the purpose of these is to provide additional insights to confirm, refute or refine proposed programme theories.[58] These are most likely to be part of Phase 3 (see below) but may also take place in the other phases if additional data is required to support programme theory development. The specific function of the interviews within a realist review means that only a small number are usually required – we anticipate no more than 12 (although the exact number is flexible and will depend on the nature and extent of gaps in the evidence). Potential interviewees may include health professionals or service users. They will be recruited via calls put out through social media and professional networks with the aim of providing new insights or testing the transferability of programme theories. Those expressing an interest will be followed up by phone/on-line call and provided with further information about project. Those who agree to proceed will be required to complete an online consent form. Interviews will be undertaken remotely, audio-recorded and analysed using a framework approach,[77] mapping the data to the CIMO configurations. Service users agreeing to an interview will be provided with a £25 Amazon voucher to recognise their time contribution.

Review Phases

Each phase of the review (with its associated search approaches) is described sequentially below, although in practice there is considerable iteration between them (see Supplemental File 1 for a flow chart of the proposed process).

Phase 1: Refining the Review Scope and Developing Initial Programme Theories

This phase aims to identify and make explicit (through CIMO configurations) an initial set of programme theories that may explain how DC-CON in maternity care can be utilised to achieve optimal outcomes, for whom and in what contexts? Given the potential variability of modality and use of DC-CON in maternity care, an important part of this process is to focus and prioritise the most important questions and outcomes.

Phase 1 involves undertaking some broad background searches to assess the breadth, depth and range of evidence available. The team will then seek to identify key papers (of any study design) on DC-CON implementation that can yield insights for programme theory

For peer review only - http://bmjopen.bmj.com/site/about/guidelines.xhtml

development. The search is iterative, utilising searches on electronic databases, grey literature sources, suggestions from stakeholders, citation tracking and reference list searching of conceptually rich index papers. This stage will include, but will not be restricted to, maternity care settings as we know from pre-protocol scoping that relevant theories have been developed regarding the implementation of DC-CON in other clinical settings.[26, 78-80] Each included paper will be scrutinised to elucidate how 'best practice' in DC-CON is defined and to identify the mechanisms through which successful consultations are purported to work in relation to different contextual configurations and population groups. Details of key theories ('candidate theories') that have been used to explain implementation mechanisms will be extracted. Excel will be used to extract the key characteristics of each paper and NVivo will be used to code the key findings related to implementation. Where possible, findings will be coded and analysed in relation to possible context, mechanism and outcome configurations, helping to generate initial ideas around relevant programme theories. The coding and analysis templates for the reviews in this stage will be developed and piloted by several members of the review team. The majority of the coding will be undertaken by one reviewer, with a second reviewer completing a random sample of approximately 20% to monitor quality and consistency. The findings of this stage will be presented using tables, figures, flow charts and narrative summaries to highlight the key features of the evidence and to describe potential programme theories.

Phase 1 includes two stakeholder workshops (at the beginning and end) to help generate initial programme theories and to refine the key focus of the review.

Phase 2: Evidence Retrieval, Review and Synthesis

The aim of Phase 2 is to determine whether the initial programme theories are supported by empirical evidence and to analyse this evidence to elaborate, refine, adjust and test the theories. This Phase continues the iterative process of literature searching, data extraction and analysis. A core focus is to search for empirical studies that can provide data with which to explore and elaborate the initial programme theories. The search strategy will be iterative, proceeding with carefully formulated searches based on sub-sets of literature constructed using terms associated with the initial programme theories (CIMO frameworks) and key concepts.[75, 81] Searches are initially broad but may then be narrowed down to focus more specifically on evidence associated with particular mechanisms. As an example, in our review, we may construct an initial search using sets of terms derived from our programme theories combined using the 'AND' Boolean operator:

- Context (from the initial programme theory) – postnatal care
- Interventions/phenomenon of interest (from the initial programme theory) – video-based consultation

A follow up search might then focus more specifically on the influence of a mechanism (e.g. trust) in relation to an outcome (e.g. positive health behaviours). In the first instance, the main focus of the search for evidence will be for literature related directly to maternity settings. However, in a realist review, the focus of analysis is the programme theory (or mechanism of action) – hence we may also draw upon wider literature to seek opportunities for transferable learning. In the example above, we might, therefore, seek evidence related

For peer review only - http://bmjopen.bmj.com/site/about/guidelines.xhtml

to ‘trust’ in the context of video consultations from other clinical settings such as primary care to confirm or refute our emerging theories.

The searches will include systematic reviews and empirical research of any study design, including service evaluation, audit and quality improvement projects. The searches will also include existing policies and practice guidelines surrounding DC-CON in maternity care in the UK. This is because the recommendations set out in policy or practice guidance rest upon implicit or explicit theoretical assumptions regarding implementation. Moreover, the inclusion of policy documents will help to ensure that governance issues are considered alongside implementation so that the review is appropriately contextualised.

As in Phase 1, search sources will include electronic databases, grey literature and expert stakeholders (see Table 2 for sources of evidence and Supplemental File 2 for an initial search strategy for OVID MEDLINE – which will be adapted for different databases). Additional search approaches will include reference list searching, citation tracking, identification of sibling papers (linked papers from a single study) as well as cluster searching[81] which involves building up rich ‘cases’ of different models of DC-CON in order to grow a cluster of related reports around named or identifiable initiatives to offer both richness and detail. Unlike a conventional systematic review search, searches in a realist review are not necessarily exhaustive but follow the principles of theoretical saturation, ceasing when programme theories are deemed to be sufficiently explained, supported or refuted by the empirical evidence.[69, 75] Likewise, additional targeted searches may be undertaken to explore new mechanisms or other aspects of programme theory that may be identified during the review.[69, 75]

Table 2: Sources of Evidence

Electronic Databases (2010-present)	Grey Literature Sources	Stakeholders
• Cochrane Central Register of Controlled Trials<br>• Cochrane Database of Systematic Reviews<br>• JBI Library<br>• MEDLINE Ovid<br>• Embase Ovid<br>• PsycINFO Ovid<br>• ASSIA Cambridge Scientific Abstracts (Applied Social Sciences Index and Abstracts)<br>• CINAHL EBCSCOhost (Cumulative Index to Nursing and Allied Health Literature)<br>• MIDIRS Ovid	• Google Scholar<br>• Websites (e.g. RCOG, RCM, RCN, NCT, NHS Trusts, NHSX, Health Foundation, WHO)<br>• Conference proceedings<br>• OpenGrey<br>• ProQuest Dissertations & Theses<br>• EThOS – British Library Electronic Theses Online	• Project Advisory Group<br>• Stakeholder groups<br>• Others (e.g. via social media requests, email list serves)

Records will be imported into EndNote and duplicates removed. Study screening and selection will be undertaken by two reviewers independently, with recourse to other team members in cases of ambiguity or disagreement. To aid transparency and consistency, once the initial programme theories have been formulated, an ‘inclusion criteria flow chart’ will be constructed in which key concepts are operationalised and against which each potential study can be assessed.[82]

For peer review only - http://bmjopen.bmj.com/site/about/guidelines.xhtml

Records will initially be screened by title and abstract. All seemingly relevant papers will then be examined in full-text and reasons for exclusion noted in a table. In line with realist methodology, records will be screened for inclusion based on relevance, rigour and richness.[58, 69] An assessment of relevance considers the extent to which a paper can directly contribute to theory building or theory testing.[69] An assessment of rigour considers whether the methods used to generate the relevant data are credible and trustworthy. Richness refers to the extent to which study findings are fully elaborated through 'thick description', grounded in the data and provide explanatory insights.[83] In a realist review, the process of quality assessment refers to the specific data that is relevant to the particular programme theory being examined rather than on the basis of a global evaluation of overall study quality.[84] Hence for each key finding, a judgment needs to be made about the plausibility and coherence of the methods used to generate it.[71] For each included paper, the team will follow the process outlined by Rycroft-Malone *et al*,[66] asking: "*is the evidence provided in this theory area good enough and relevant enough to be included?*". These judgements will be articulated and recorded for each study as part of the screening and data extraction process.

Data will be extracted from the included studies in two ways. First, information about study characteristics will be extracted into a summary table (as is the case with a conventional systematic review). This will include information on features such as study setting, design, methods, details of intervention modality and technology, participants, outcomes and stage of maternity care pathway. Second, a bespoke data extraction form will be developed based on the initial programme theories, and, as noted above, will include sections in which to note assessments of relevance, richness and rigour. Theory-based data extraction enables the coding and charting of relevant data so that elements of the theory can become populated to investigate what works, for whom, how and in what contexts. The analytical process involves both deductive and inductive coding. Deductive coding will involve extracting data that appears to be directly related to aspects of the programme theory. Where it is possible to make relevant inferences, the data will also be coded in relation to contexts, mechanisms or outcomes. However, the evidence may also reveal new contexts, mechanisms or outcomes which will be identified and coded inductively.

The data extraction templates and associated analytical process will be developed collaboratively amongst the core research team and piloted extensively. Once the team feel they have achieved a clear and consistent approach, the remaining data extraction will be undertaken by one reviewer, with a second reviewer checking approximately 20%. The outputs of this stage will be a set of evidence tables. There will be one overarching table representing the key characteristics of all the studies included in the review. There will then be a series of tables organised to represent the different bodies of literature and findings related to each initial programme theory. Thus, each theory area will have its own evidence table.

The process of data analysis will be ongoing and iterative. The evidence will be reviewed within and across the theory areas to explore how it builds upon, refutes or provides alternative explanations for the initial CIMO configurations. The analytical process will involve asking questions such as: "*What does this evidence suggest about this aspect of our theory? Does it support it? Does it disprove it? Does it suggest an amendment to it*?"[69] This analytic process involves both abductive and retroductive reasoning – i.e. making new

For peer review only - http://bmjopen.bmj.com/site/about/guidelines.xhtml

observations from the evidence, inferring plausible explanations related to the programme theory, seeking to understand the cause of perceived events beyond what can be observed and seeking to identify over-arching patterns. Wong *et al*[69] propose a series of 'conceptual tools' (derived from Pawson, 2005[59]) which will be employed by the review team in this complex process, as indicated in Table 3 below.

Table 3: Analytic Process of a Realist Review

- Juxtaposition of sources of evidence—for example, where evidence about outcomes in one study allows insights into evidence about outcomes in another study;
- Reconciling of sources of evidence—where results differ in comparable circumstances, these will be examined further to find possible reasons for the different results;
- Adjudication of sources of evidence—based on methodological strengths or weaknesses;
- Consolidation of sources of evidence—where outcomes differ in particular contexts, an explanation will be constructed on how and why these outcomes occur differently;
- Situating sources of evidence—when outcomes are different in particular contexts, a possible explanation will be developed as to why they differ

Our stakeholder groups will continue to be consulted at different points to seek their views and test out new ideas.

An important feature of the analysis will be to explore the issue of temporality and sustainability within the evidence. For example, it might be expected that evidence on Covid-19-related DC-CON implementation might include different experiences depending upon the stage of the pandemic and the length of time that staff and service users have had to adjust to changes in service delivery models. Likewise, even in non-Covid-related studies, experiences with new technologies vary depending upon the length of time since introduction, with many studies focusing upon the early stages of implementation rather than exploring how and why technologies become embedded, normalised or sustainable.[80, 85]

In this project, we will endeavour to take temporality into account in two ways. The first, relates to evidence that is specifically concerned with changes made during the Covid-19 pandemic. Here, we will ensure that our data extraction template explicitly acknowledges the time period of data collection, extracting and mapping data to the stages of the pandemic and then transparently considering these stages as part of the analysis. Likewise, working with our advisory and stakeholder groups, we will attempt to develop a clearer picture of how digital consultations were initially introduced in different settings and how their use and implementation may have adapted and changed as the pandemic progressed. The second issue relates to the fact that not all of the evidence that we anticipate including in the review will come from the pandemic context. We will endeavour to include temporality as an aspect of our analysis for all the evidence by explicitly considering the stage of implementation of an innovation that is reported and by considering data related to change and adaptation over time (where reported). We plan to draw upon concepts from the Dynamic Sustainability Framework[86] to help 'sensitise' the team to issues of temporality. The framework focuses attention to the constantly changing evidence-base, the multi-level context in which interventions are delivered, and the broader ecological system within which the maternity care exists and operates.

For peer review only - http://bmjopen.bmj.com/site/about/guidelines.xhtml

The output of Phase 2 will be a comprehensive set of evidence tables and refined programme theories linked to associated sets of working papers organised by DC-CON type and purpose in relation to different points in the maternity care pathway.

Phase 3: Test and Refine Programme Theories (Validation)

The purpose of this Phase is to test and further refine the programme theories. This is done in three interlinked ways.

First, as part of an ongoing recursive approach, new literature searches will be undertaken to find, test and empirically explore any new theories identified during the review process.[75] This may also include a need to re-visit and re-code previously identified papers. As noted by Booth et al,[75] this stage may involve searches for specific named theories that have been identified in Phase 2 and that are considered worthy of further empirical exploration.

Second, further in-depth consultation/workshops with stakeholders will be undertaken to test and refine proposed theories. Our stakeholder groups will be asked to consider the proposed theories in relation to their own experiences, paying particular attention to clarifying the key mechanisms that are required to produce desired outcomes and to identify the key links between these mechanisms and different contexts. This process will help to identify 'gaps' that remain in our understanding and to clarify the essential elements of intervention and context that need to be place to ensure that the appropriate mechanisms can be activated.

Third, as described above, we will undertake a limited number of key informant interviews to test aspects of the programme theories and to evaluate their plausibility, credibility and transferability.[58]

The output of this Phase will be a theoretically-grounded explanatory framework for safe, appropriate and acceptable DC-CON in maternity care that can be used to guide intervention development, policy and practice.

Phase 4: Development of Actionable Recommendations

The purpose of this Phase is to generate actionable recommendations from the review. We will do this by holding an on-line webinar to which a much larger group of stakeholders will be invited. The purpose of this webinar will be to share and 'sense-check' the review findings and to solicit suggestions for recommendations, actions and strategies for dissemination. In addition, we will hold further meetings to discuss, develop and finalise recommendations appropriate to a range of constituents (researchers, commissioners, service leads, professionals, women, community organisations) and feasible for implementation in a UK NHS context.

ETHICS AND DISSEMINATION

Ethical approval for the key informant interviews has been obtained from the University of Nottingham Faculty of Medicine and Health Sciences Research Ethics Committee –

For peer review only - http://bmjopen.bmj.com/site/about/guidelines.xhtml

reference no. FMHS 426-1221. Formal ethical approval for the stakeholder involvement/consultations is not required, however the principles of research integrity and informed consent will be followed at all times.

Dissemination strategies will be informed through ongoing engagement with our stakeholder groups. Key strategies will include academic papers, conference presentations, development of an online educational resource for health professionals and short papers in 'practice focused' journals.

AUTHOR'S CONTRIBUTIONS

The original protocol was developed with extensive conceptualisation and written input from all authors. CE prepared the protocol as a manuscript for publication. All authors have read, commented on and approved the final manuscript. AB contributed inputs on realist methodology. MC provided inputs on the search strategy. ST provided inputs on implementation science issues. KE, HS, BN and NJ contributed sections on maternity policy and practice. CS provided guidance on conceptualising issues related to PPI and stakeholder involvement. GC contributed to the revisions required following peer review and drafted the response to reviewers.

FUNDING STATEMENT

This work is supported by the UK National Institute for Health Research (NIHR), Health Services Delivery Research Programme, grant number: NIHR134535.

DISCLAIMER

The views expressed are those of the authors and not necessarily those of the NIHR or the UK Department of Health and Social Care.

COMPETING INTERESTS STATEMENT

None to declare

WORD COUNT

5,490

For peer review only - http://bmjopen.bmj.com/site/about/guidelines.xhtml

REFERENCES

1. National Maternity Review. Better Births: Improving Outcomes of Maternity Services in England: A Five Year Forward View for Maternity Care. 2016. Available from: https://www.england.nhs.uk/wp-content/uploads/2016/02/national-maternity-review-report.pdf (accessed 09/02/21)

2. NHS England. Maternity Transformation Programme. Available from: https://www.england.nhs.uk/mat-transformation/ (accessed 09/02/21)

3. NHS England. Better Births Four Years On: A Review of Progress 2020. Available from: https://www.england.nhs.uk/wp-content/uploads/2020/03/better-births-four-years-on-progress-report.pdf (accessed 09/02/21)

4. NHS Digital. Digital Maternity: Harnessing Digital Technology in Maternity Services. Contract No.: 09/02/21. Available from: https://digital.nhs.uk/services/digital-maternity-programme (accessed 25/01/22)

5. NHS Digital. Maternity DMA Report: Digital Maturity Assessment of Maternity Services in England. 2018. Available from: https://www.england.nhs.uk/wp-content/uploads/2018/11/national-maternity-dma-report.pdf (accessed 11/03/21)

6. Renfrew MJ, Cheyne H, Craig J, *et al.* Sustaining quality midwifery care in a pandemic and beyond. *Midwifery*. 2020;**88**:102759-59. doi:10.1016/j.midw.2020.102759

7. Jardine J, Relph S, Magee LA, *et al.* Maternity services in the UK during the coronavirus disease 2019 pandemic: a national survey of modifications to standard care. *BJOG*. 2020. doi:10.1111/1471-0528.16547

8. Fernandez Turienzo C, Newburn M, Agyepong A, *et al.* Addressing inequities in maternal health among women living in communities of social disadvantage and ethnic diversity. *BMC Public Health*. 2021;**21**:176. doi:10.1186/s12889-021-10182-4

9. Hinton L, Kuberska K, Dakin F, *et al.* Creating equitable remote antenatal care: the importance of inclusion. *BMJ Opinion*. 2021.

10. Royal College of Midwives. Virtual consultations: guidance on appropriate application for virtual consultations and practical tips for effective use (V2.3). 2020. Available from: https://www.rcm.org.uk/media/4596/virtual-consultations-v23-revised-17-december-2021-review-25-january-2021.pdf (accessed 05/02/21)

11. Royal College of Midwives, Royal College of Obstetricians & Gynaecologists. Guidance for Antenatal and Postnatal Services in the evolving Coronavirus (COVID-19) pandemic (Version 3). 2020. Available from: https://www.rcog.org.uk/globalassets/documents/guidelines/2020-10-21-guidance-for-antenatal-and-postnatal-services-in-the-evolving-coronavirus-covid-19-pandemic-v3.pdf (accessed 05/02/21)

12. Saad M, Chan S, Nguyen L, *et al.* Patient perceptions of the benefits and barriers of virtual postnatal care: a qualitative study. *BMC Pregnancy and Childbirth*. 2021;**21**:543-43. doi:10.1186/s12884-021-03999-9

For peer review only - http://bmjopen.bmj.com/site/about/guidelines.xhtml

13. Riley V, Ellis N, Mackay L, *et al.* The impact of COVID-19 restrictions on women's pregnancy and postpartum experience in England: A qualitative exploration. *Midwifery*. 2021;**101**:103061-61. doi:10.1016/j.midw.2021.103061

14. John JR, Curry G, Cunningham-Burley S. Exploring ethnic minority women's experiences of maternity care during the SARS-CoV-2 pandemic: a qualitative study. *BMJ Open*. 2021;**11**:e050666. doi:10.1136/bmjopen-2021-050666

15. McKinlay AR, Fancourt D, Burton A. Factors affecting the mental health of pregnant women using UK maternity services during the COVID-19 pandemic: A qualitative interview study. *medRxiv*. 2021:2021.10.20.21265279. doi:10.1101/2021.10.20.21265279

16. Tavener CR, Kyriacou C, Elmascri I, *et al.* Rapid introduction of virtual consultation in a hospital-based Consultant-led Antenatal Clinic to minimise exposure of pregnant women to COVID-19. *BMJ Open Quality*. 2022;**11**:e001622. doi:10.1136/bmjoq-2021-001622

17. Brislane Á, Larkin F, Jones H, *et al.* Access to and Quality of Healthcare for Pregnant and Postpartum Women During the COVID-19 Pandemic. *Frontiers in Global Women's Health*. 2021;**2**. doi:10.3389/fgwh.2021.628625

18. Quinn LM, Olajide O, Green M, *et al.* Patient and Professional Experiences With Virtual Antenatal Clinics During the COVID-19 Pandemic in a UK Tertiary Obstetric Hospital: Questionnaire Study. *J Med Internet Res*. 2021;**23**:e25549. doi:10.2196/25549

19. Sanders J, Blaylock R, WRISK Study Team. Users' experiences of COVID-19 maternity service changes (Version 2). 2020. Available from: https://wrisk.org/wp-content/uploads/2021/02/WRISK-COVID-Report_Dec20_V2.pdf (accessed 05/02/21)

20. Gülmezoglu AM, Ammerdorffer A, Narasimhan M, *et al.* Self-care and remote care during pregnancy: a new paradigm? *Health Research Policy and Systems*. 2020;**18**:107. doi:10.1186/s12961-020-00627-4

21. Royal College of Midwives. Digital Technology in Maternity Care: A Position Statement 2021. Available from: https://www.rcm.org.uk/media/4789/rcm_position-statement_digital-technologies_final.pdf (accessed 16/03/21)

22. The Health Foundation. Securing a Positive Healthcare Technology Legacy from Covid-19. 2021. Available from: https://www.health.org.uk/publications/long-reads/securing-a-positive-health-care-technology-legacy-from-covid-19 (accessed 08-04-21)

23. Bick D, Cheyne H, Chang Y-S, *et al.* Maternal postnatal health during the COVID-19 pandemic: Vigilance is needed. *Midwifery*. 2020;**88**:102781. doi:https://doi.org/10.1016/j.midw.2020.102781

24. NHS England & NHS Improvement. Equity and equality: Guidance for local maternity systems. 2021. Available from: https://www.england.nhs.uk/wp-content/uploads/2021/09/C0734-equity-and-equality-guidance-for-local-maternity-systems.pdf (accessed 07/02/22)

25. World Health Organisation. Classification of Digital Health Interventions v1.0. 2017. Available from: https://apps.who.int/iris/bitstream/handle/10665/260480/WHO-RHR-18.06-eng.pdf;jsessionid=1DA9EAD9506E29BB5E1ED23FB797EF9F?sequence=1 (accessed 16/03/21)

For peer review only - http://bmjopen.bmj.com/site/about/guidelines.xhtml

26. Griffiths FE, Armoiry X, Atherton H*, et al.* The role of digital communication in patient-clinician communication for NHS providers of specialist clinical services for young people [the Long-term conditions Young people Networked Communication (LYNC) study]: a mixed-methods study. *Health Services and Delivery Research*. 2018;**6**. doi:https://doi.org/10.3310/hsdr06090

27. Lee Y, Cho S. Technology-Supported Interventions for Pregnant Women: A Systematic Review. *Comput Inform Nurs*. 2019;**37**:501-12. doi:10.1097/cin.0000000000000535

28. van den Heuvel JF, Groenhof TK, Veerbeek JH*, et al.* eHealth as the Next-Generation Perinatal Care: An Overview of the Literature. *J Med Internet Res*. 2018;**20**:e202. doi:10.2196/jmir.9262

29. DeNicola N, Grossman D, Marko K*, et al.* Telehealth Interventions to Improve Obstetric and Gynecologic Health Outcomes: A Systematic Review. *Obstet Gynecol*. 2020;**135**:371-82. doi:10.1097/aog.0000000000003646

30. Lavender T, Richens Y, Milan SJ*, et al.* Telephone support for women during pregnancy and the first six weeks postpartum. *Cochrane Database of Systematic Reviews*. 2013. doi:10.1002/14651858.CD009338.pub2

31. Kapinos K, Kotzias V, Bogen D*, et al.* The Use of and Experiences With Telelactation Among Rural Breastfeeding Mothers: Secondary Analysis of a Randomized Controlled Trial. *J Med Internet Res*. 2019;**21**:e13967. doi:10.2196/13967

32. Habibi MF, Nicklas J, Spence M*, et al.* Remote lactation consultation: a qualitative study of maternal response to experience and recommendations for survey development. *J Hum Lact*. 2012;**28**:211-7. doi:10.1177/0890334411432716

33. Ames HMR, Glenton C, Lewin S*, et al.* Clients' perceptions and experiences of targeted digital communication accessible via mobile devices for reproductive, maternal, newborn, child, and adolescent health: a qualitative evidence synthesis. *Cochrane Database of Systematic Reviews*. 2019. doi:10.1002/14651858.CD013447

34. Bailey CM, Newton JM, Hall HG. Telephone triage and midwifery: A scoping review. *Women Birth*. 2018;**31**:414-21. doi:10.1016/j.wombi.2017.12.002

35. Jeganathan S, Prasannan L, Blitz MJ*, et al.* Adherence and acceptability of telehealth appointments for high-risk obstetrical patients during the coronavirus disease 2019 pandemic. *Am J Obstet Gynecol MFM*. 2020;**2**:100233. doi:10.1016/j.ajogmf.2020.100233

36. Khalil C. Understanding the Adoption and Diffusion of a Telemonitoring Solution in Gestational Diabetes Mellitus: Qualitative Study. *JMIR Diabetes*. 2019;**4**:e13661. doi:10.2196/13661

37. Butler Tobah YS, LeBlanc A, Branda ME*, et al.* Randomized comparison of a reduced-visit prenatal care model enhanced with remote monitoring. *Am J Obstet Gynecol*. 2019;**221**:638.e1-38.e8. doi:10.1016/j.ajog.2019.06.034

38. Marko KI, Ganju N, Brown J*, et al.* Remote Prenatal Care Monitoring With Digital Health Tools Can Reduce Visit Frequency While Improving Satisfaction. *Obstetrics & Gynecology*. 2016;**127**:1S.

For peer review only - http://bmjopen.bmj.com/site/about/guidelines.xhtml

39. Panda S, O'Malley D, Barry P*, et al.* Women's views and experiences of maternity care during COVID-19 in Ireland: A qualitative descriptive study. *Midwifery*. 2021;**103**:103092. doi:https://doi.org/10.1016/j.midw.2021.103092

40. Seguranyes G, Costa D, Fuentelsaz-Gallego C*, et al.* Efficacy of a videoconferencing intervention compared with standard postnatal care at primary care health centres in Catalonia. *Midwifery*. 2014;**30**:764-71. doi:https://doi.org/10.1016/j.midw.2013.08.004

41. Feroz A, Perveen S, Aftab W. Role of mHealth applications for improving antenatal and postnatal care in low and middle income countries: a systematic review. *BMC Health Services Research*. 2017;**17**:704. doi:10.1186/s12913-017-2664-7

42. Linden K, Domgren N, Zaigham M*, et al.* Being in the shadow of the unknown — Swedish women's lived experiences of pregnancy during the COVID-19 pandemic, a phenomenological study. *Women and Birth*. 2021. doi:https://doi.org/10.1016/j.wombi.2021.09.007

43. Karavadra B, Stockl A, Prosser-Snelling E*, et al.* Women's perceptions of COVID-19 and their healthcare experiences: a qualitative thematic analysis of a national survey of pregnant women in the United Kingdom. *BMC Pregnancy and Childbirth*. 2020;**20**:600. doi:10.1186/s12884-020-03283-2

44. Penny RA, Bradford NK, Langbecker D. Registered nurse and midwife experiences of using videoconferencing in practice: A systematic review of qualitative studies. *J Clin Nurs*. 2018;**27**:e739-e52. doi:10.1111/jocn.14175

45. Harrison TN, Sacks DA, Parry C*, et al.* Acceptability of Virtual Prenatal Visits for Women with Gestational Diabetes. *Women's Health Issues*. 2017;**27**:351-55. doi:https://doi.org/10.1016/j.whi.2016.12.009

46. Liu CH, Goyal D, Mittal L*, et al.* Patient Satisfaction with Virtual-Based Prenatal Care: Implications after the COVID-19 Pandemic. *Matern Child Health J*. 2021;**25**:1735-43. doi:10.1007/s10995-021-03211-6

47. Kolker S, Biringer A, Bytautas J*, et al.* Pregnant during the COVID-19 pandemic: an exploration of patients' lived experiences. *BMC Pregnancy and Childbirth*. 2021;**21**:851-51. doi:10.1186/s12884-021-04337-9

48. Atmuri K, Sarkar M, Obudu E*, et al.* Perspectives of pregnant women during the COVID-19 pandemic: A qualitative study. *Women and Birth*. 2021. doi:https://doi.org/10.1016/j.wombi.2021.03.008

49. Keating N, Dempsey B, Corcoran S*, et al.* Women's Experience of Pregnancy and Birth during the Covid-19 Pandemic: A Qualitative Study. Research Square; 2020.

50. Altman MR, Gavin AR, Eagen-Torkko MK*, et al.* Where the System Failed: The COVID-19 Pandemic's Impact on Pregnancy and Birth Care. *Global Qualitative Nursing Research*. 2021;**8**:23333936211006397. doi:10.1177/23333936211006397

51. Birthrights. Systemic Racism, Not Broken Bodies. 2022. Available from: https://www.birthrights.org.uk/campaigns-research/racial-injustice/ (accessed 01-06-22)

52. Hinton L, Dakin FH, Kuberska K*, et al.* Quality framework for remote antenatal care: qualitative study with women, healthcare professionals and system-level stakeholders. *BMJ Quality & Safety*. 2022. doi:10.1136/bmjqs-2021-014329

For peer review only - http://bmjopen.bmj.com/site/about/guidelines.xhtml

53. Huxley CJ, Atherton H, Watkins JA*, et al.* Digital communication between clinician and patient and the impact on marginalised groups: a realist review in general practice. *British Journal of General Practice*. 2015;**65**:e813. doi:10.3399/bjgp15X687853

54. Rayment-Jones H, Harris J, Harden A*, et al.* How do women with social risk factors experience United Kingdom maternity care? A realist synthesis. *Birth*. 2019;**46**:461-74. doi:10.1111/birt.12446

55. Rayment-Jones H, Harris J, Harden A*, et al.* Project20: interpreter services for pregnant women with social risk factors in England: what works, for whom, in what circumstances, and how? *Int J Equity Health*. 2021;**20**:233. doi:10.1186/s12939-021-01570-8

56. Wu KK, Lopez C, Nichols M. Virtual Visits in Prenatal Care: An Integrative Review. *J Midwifery Womens Health*. 2022;**67**:39-52. doi:10.1111/jmwh.13284

57. Fryer K, Delgado A, Foti T*, et al.* Implementation of Obstetric Telehealth During COVID-19 and Beyond. *Matern Child Health J*. 2020;**24**:1104-10. doi:10.1007/s10995-020-02967-7

58. Pawson R, Greenhalgh T, Harvey G*, et al.* Realist review--a new method of systematic review designed for complex policy interventions. *J Health Serv Res Policy*. 2005;**10 Suppl 1**:21-34. doi:10.1258/1355819054308530

59. Pawson R. Evidence Based Policy: A Realist Perspective. London: Sage; 2006.

60. Bhaskar R. A Realist Theory of Science. Leeds: Leeds Books; 2008.

61. Archer M. Realist Social Theory: The Morphogenic Approach. Cambridge: Cambridge University Press; 1995.

62. Emmel N, Manzano A, Dalkin S*, et al.* Doing Realist Research. London: Sage 2018.

63. Bauer MS, Damschroder L, Hagedorn H*, et al.* An introduction to implementation science for the non-specialist. *BMC Psychology*. 2015;**3**:32. doi:10.1186/s40359-015-0089-9

64. Craig P, Dieppe P, Macintyre S*, et al.* Developing and evaluating complex interventions: the new Medical Research Council guidance. *BMJ*. 2008;**337**:a1655. doi:10.1136/bmj.a1655

65. Pawson R, Tilley N. Realistic Evaluation. London: Sage; 1997.

66. Rycroft-Malone J, McCormack B, Hutchinson AM*, et al.* Realist synthesis: illustrating the method for implementation research. *Implementation Science*. 2012;**7**:33. doi:10.1186/1748-5908-7-33

67. Dalkin SM, Greenhalgh J, Jones D*, et al.* What's in a mechanism? Development of a key concept in realist evaluation. *Implementation Science*. 2015;**10**:49. doi:10.1186/s13012-015-0237-x

68. Shaw J, Gray CS, Baker GR*, et al.* Mechanisms, contexts and points of contention: operationalizing realist-informed research for complex health interventions. *BMC Medical Research Methodology*. 2018;**18**:178. doi:10.1186/s12874-018-0641-4

For peer review only - http://bmjopen.bmj.com/site/about/guidelines.xhtml

69. Wong G, Westthorp G, Pawson R*, et al.* Realist Synthesis: RAMESES Training Materials. 2013. Available from: https://www.ramesesproject.org/media/Realist_reviews_training_materials.pdf (accessed 02-02-21)

70. Hunter R, Gorely T, Beattie M*, et al.* Realist review. *International Review of Sport and Exercise Psychology*. 2022:1-24. doi:10.1080/1750984X.2021.1969674

71. Wong G, Greenhalgh T, Westhorp G*, et al.* RAMESES publication standards: realist syntheses. *BMC Medicine*. 2013;**11**:21. doi:10.1186/1741-7015-11-21

72. NIHR. UK Standards for Public Involvement in Research. Available from: https://sites.google.com/nihr.ac.uk/pi-standards/home?authuser=0 (accessed 21-09-21)

73. Staniszewska S, Brett J, Simera I*, et al.* GRIPP2 reporting checklists: tools to improve reporting of patient and public involvement in research. *BMJ*. 2017;**358**:j3453. doi:10.1136/bmj.j3453

74. Evans K, Janiszewski H, Evans C*, et al.* Establishing information needs and research priorities in response to the Covid-19 pandemic in the local maternity setting. *Midwifery*. 2021;**95**:102922. doi:10.1016/j.midw.2021.102922

75. Booth A, Wright J, Briscoe S. Scoping and searching to support realist approaches In: Emmel N, Greenhalgh J, Manzano A*et al*, editors. Doing Realist Research. London: Sage Publications; 2018. p. 147-66. (accessed

76. Booth A, Briscoe S, Wright JM. The "realist search": A systematic scoping review of current practice and reporting. *Res Synth Methods*. 2020;**11**:14-35. doi:10.1002/jrsm.1386

77. Gale NK, Heath G, Cameron E*, et al.* Using the framework method for the analysis of qualitative data in multi-disciplinary health research. *BMC Medical Research Methodology*. 2013;**13**:117. doi:10.1186/1471-2288-13-117

78. Greenhalgh T, Shaw S, Wherton J*, et al.* Real-World Implementation of Video Outpatient Consultations at Macro, Meso, and Micro Levels: Mixed-Method Study. *Journal of Medical Internet Research*. 2018;**20**:e150-e50. doi:10.2196/jmir.9897

79. Vassilev I, Rowsell A, Pope C*, et al.* Assessing the implementability of telehealth interventions for self-management support: a realist review. *Implement Sci*. 2015;**10**:59. doi:10.1186/s13012-015-0238-9

80. May C, Finch T, Mair F*, et al.* Understanding the implementation of complex interventions in health care: the normalization process model. *BMC Health Serv Res*. 2007;**7**:148. doi:10.1186/1472-6963-7-148

81. Booth A, Harris J, Croot E*, et al.* Towards a methodology for cluster searching to provide conceptual and contextual "richness" for systematic reviews of complex interventions: case study (CLUSTER). *BMC Med Res Methodol*. 2013;**13**:118. doi:10.1186/1471-2288-13-118

82. Williams L, Rycroft-Malone J, Burton CR*, et al.* Improving skills and care standards in the support workforce for older people: a realist synthesis of workforce development interventions. *BMJ Open*. 2016;**6**:e011964. doi:10.1136/bmjopen-2016-011964

For peer review only - http://bmjopen.bmj.com/site/about/guidelines.xhtml

83. Popay J, Rogers A, Williams G. Rationale and standards for the systematic review of qualitative literature in health services research. *Qual Health Res*. 1998;**8**:341-51. doi:10.1177/104973239800800305

84. Pawson R. Digging for nuggets: how 'bad' research can yield 'good' evidence. *International Journal of Social Research Methodology*. 2006;**9**:127-42.

85. May C. Towards a general theory of implementation. *Implementation Science*. 2013;**8**:18. doi:10.1186/1748-5908-8-18

86. Chambers DA, Glasgow RE, Stange KC. The dynamic sustainability framework: addressing the paradox of sustainment amid ongoing change. *Implementation Science*. 2013;**8**:117. doi:10.1186/1748-5908-8-117

For peer review only

Supplemental File 1: Digital Clinical Consultation in Maternity Care: Realist Review Protocol Process Flowchart

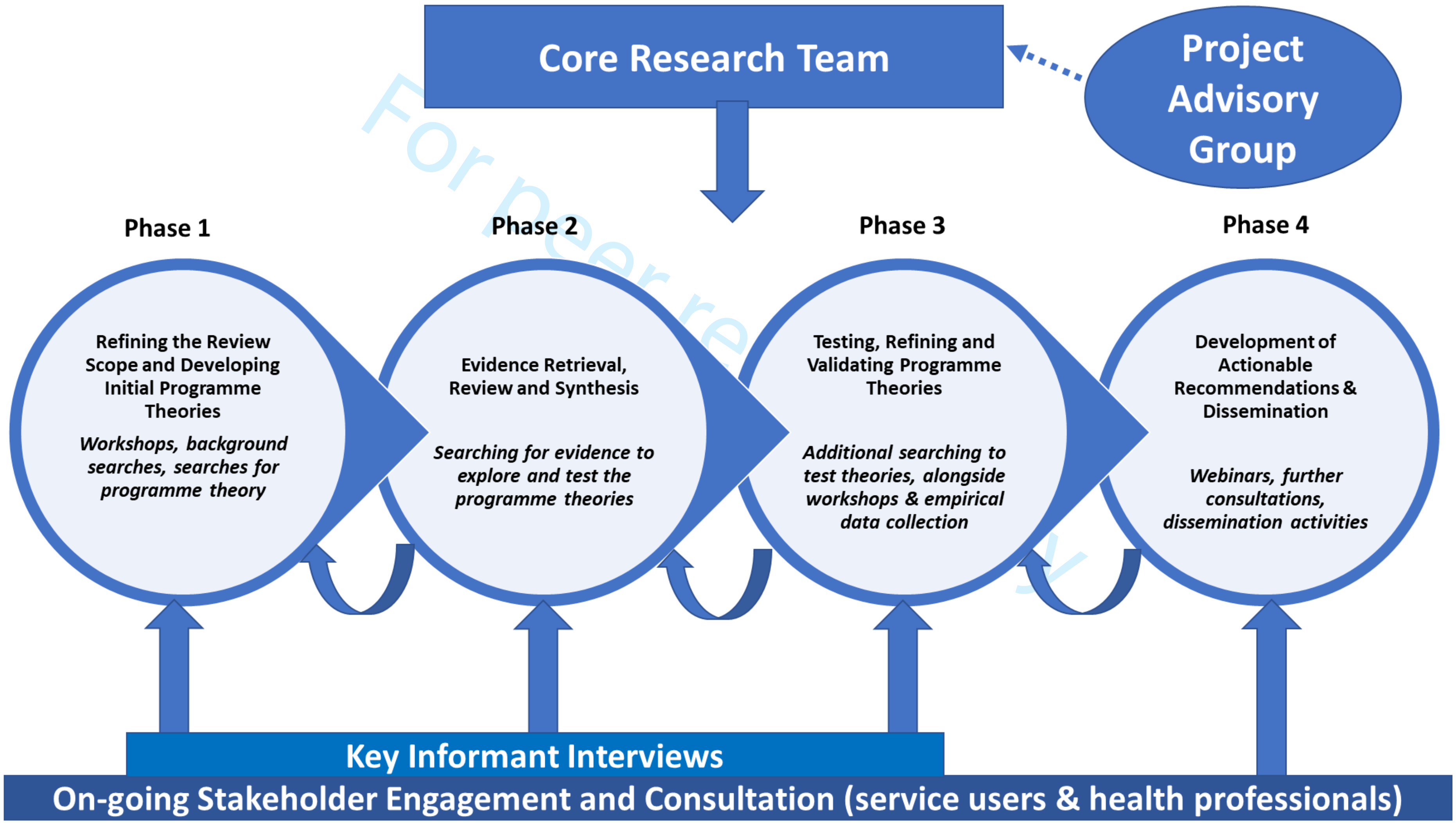

Supplemental File No. 2: Initial Search Strategy

Database: Ovid MEDLINE(R) and Epub Ahead of Print, In-Process, In-Data-Review & Other Non-Indexed Citations, Daily and Versions(R) <1946 to January 27, 2022>

#	Query	Results from 28 Jan 2022
1	exp Telemedicine/	38,848
2	remote consultation/ or videoconferencing/	7,285
3	(telemedicine or tele-medicine or telecare or tele-care or telehealth or tele-health).mp. [mp=title, abstract, original title, name of substance word, subject heading word, floating sub-heading word, keyword heading word, organism supplementary concept word, protocol supplementary concept word, rare disease supplementary concept word, unique identifier, synonyms]	44,080
4	((remote* or virtual* or online or on-line or digital*) adj3 (consultation* or appointment* or meet*)).mp.	8,514
5	(videoconferenc* or video-conferenc* or teleconferenc* or tele-conferenc* or zoom or facetime or face-time).mp.	8,842
6	1 or 2 or 3 or 4 or 5	56,928
7	exp Maternal Health Services/	54,815
8	exp Prenatal Care/ or exp Midwifery/ or exp Pregnancy/ or exp Obstetrics/	972,055
9	(matern* or pregnan* or prenatal or pre-natal or antenatal or ante-natal or perinatal or peri-natal or postnatal or post-natal or postpartum or post-partum or breastfeed* or breast feed* or midwi* or obstetric*).mp.	1,408,667
10	7 or 8 or 9	1,420,532
11	6 and 10	1,835
12	Primary Health Care/	86,523
13	general practice/ or family practice/	77,081
14	(primary care or general practice or GP or family doctor* or family physician*).mp.	225,104
15	exp Endocrinologists/	222
16	exp Cardiologists/	986
17	(endocrinologist* or cardiologist*).mp.	21,716
18	12 or 13 or 14 or 15 or 16 or 17	309,535
19	6 and 18	4,484
20	19 not 11	4,360
21	(framework* or model* or theor* or concept*).mp.	5,371,555
22	limit 21 to yr="2010 -Current"	3,213,643
23	11 and 22	407
24	20 and 22	1,060

For peer review only
